# Effects of Compression on Extracellular Matrix Synthesis by Chondrocytes and Chondrosarcoma Cells

**DOI:** 10.1177/19476035261460455

**Published:** 2026-07-17

**Authors:** Carlo Alberto Paggi, Isa Porsul, Séverine Le Gac, Marcel Karperien

**Affiliations:** 1 Department of Developmental BioEngineering, TechMed Centre; 2 Applied Microfluidics for BioEngineering Research, MESA + Institute for Nanotechnology & TechMed Centre, University of Twente, Enschede, The Netherlands; 3 Organ-on-Chip Centre, 3230 University of Twente, Enschede, The Netherlands

**Keywords:** cartilage mechanobiology, chondrocytes, chondrosarcoma, mechanical loading, extracellular matrix

## Abstract

**Objective:**

Mechanical loading is critical for chondrocyte function and cartilage matrix production, yet its effects on clustered chondrocytes in end-stage osteoarthritis and on chondrosarcoma cells remain poorly understood. This study investigated how cyclic compression influences chondrocytes and chondrosarcoma cells cultured either as single cells or as micromasses.

**Methods:**

An organ-on-chip platform was used to apply cyclic compression to chondrocytes and chondrosarcoma cells embedded in agarose as single cells or micromasses mimicking chondrocyte clusters. Deformation during loading was assessed, and after four days of stimulation, gene and protein expression of cartilage markers and extracellular matrix components were analyzed.

**Results:**

Chondrocyte micromasses deformed less than single cells and showed behavior resembling chondrosarcoma cells and their micromasses. All four culture models exhibited distinct mechanobiological responses after four days of compression. Mechanical loading promoted collagen I reorganization in micromasses and increased **ACAN** and **COL2A1** expression, with enhanced matrix deposition aligned with the loading direction in one chondrocyte donor and one chondrosarcoma cell line. Semi-quantitative imaging suggested trends toward ECM deposition along the loading axis in some micromass conditions.

**Conclusion:**

In this agarose-based organ-on-chip proof-of-concept model, cellular organization was associated with distinct responses to cyclic compression. These findings should be interpreted as model-specific observations from one OA donor and one chondrosarcoma cell line, and require validation in additional donors, cell lines, matrices, and loading conditions before broader conclusions can be drawn.

## Graphical abstract

**Graphical Abstract:**
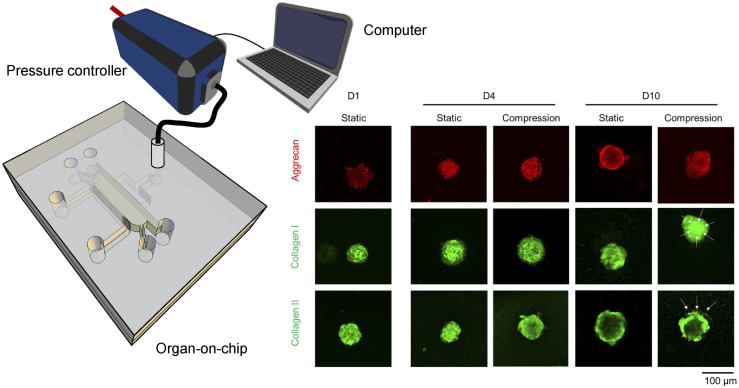

## 1. Introduction

Articular cartilage, which covers the distal ends of bones in the joint, comprises one specialized cell type, the chondrocytes.^1^ In native tissue, chondrocytes are surrounded by a pericellular matrix, which protects them from the mechanical load exerted on the tissue during movement while acting as a signal transducer of this mechanical stimulation.^2^ Damage to the superficial layers of the cartilage lead, in most cases, to a slow but progressive degradation of the entire tissue due to its low self-healing capacity. In osteoarthritis (OA) deterioration of the cartilage superficial layers affects the chondrocytes that adopt a more hypertrophic phenotype.^3^ As a result, the extracellular matrix (ECM) in the entire tissue undergoes structural re-arrangement and chondrocytes start proliferating, yielding micromasses that are enriched in collagen I and that progressively acquire a more osteogenic phenotype.^4^ However, little is known about this clustering effect and its impact on the cellular responsiveness to mechanical load and how this translates in the production of COL2A1 in the extracellular matrix and collagen VI in the pericellular matrix.

Chondrosarcoma is a bone tumour accounting for 20% of all bone tumours, that exhibits cartilage-like characteristics.^5^ About 80-85% of the chondrosarcomas are “conventional” or primary chondrosarcomas, in which hyaline cartilage is formed in the medullar cavity of bones like the pelvis, femur and humerus.^6^ Surgery is the primary treatment for chondrosarcomas.^7^ Yet, depending on their location, surgical excision is not always possible, in which cases other treatments are demanded. However, due to its cartilage-like characteristics, chondrosarcoma is resistant to both chemotherapy and radiation.^6^ Resistance to chemotherapy can specifically be accounted for by the slow cell proliferation, the absence of blood vessels, the presence of a dense ECM network and the overexpression of multidrug resistance (MDR) proteins such as MDR1.^8^

Chondrocytes and chondrosarcoma cells are widely used to study cartilage matrix production,^9^ in spite of the striking differences they present. Chondrosarcomas, for example, are surrounded by abundant ECM varying both in size and shape,^5,9^ and often comprise cells with enlarged and even binucleated nuclei.^10^ Calcified areas can also be found in those tissues, indicating the presence of a pre-existing enchondroma which is a non-cancerous bone tumour.^9^ In contrast to articular chondrocytes, for which mechanical loading is crucial for ensuring proper ECM production,^11^ this is less clear for chondrosarcomas. This is relevant since their embedding in the medular cavity of the bone constitutes a completely different biomechanical environment compared to the articular cartilage. Collectively, these observations raise the question whether mechanical stimulation is involved in the extensive ECM production by chondrosarcoma cells.

Mechanical stimulation on articular chondrocytes has been widely investigated.^12,13^ However, the use of large tissue-engineered constructs or explants prevents real-time visualization of individual cell responses. Organ-on-chip technologies are progressively being adopted to investigate cellular response towards mechanical stimulation. Due to the important nature of this parameter in articular cartilage, various models have been developed to exert different types of mechanical stimuli onto chondrocytes.^14,15^ One example being the cartilage-on-chip of Occhetta et al. where physiological and hyper-physiological mechanical stimuli were applied to a 3D construct.^16^ Another strategy used shear stress induced by flow on equine chondrocytes which promoted better cartilage matrix production.^17^

Here, we aimed to address the following proof-of-concept questions in a controlled agarose-based organ-on-chip model: (i) How does mechanical compression affect ECM production of chondrocyte clusters in comparison to single chondrocytes? These clusters mimic the start of chondrogenesis during endochondral ossification and clustering of chondrocytes is frequently used to stimulate cartilage matrix formation in vitro.^18^ Alternatively, chondrocyte clusters could serve as a model for chondrocyte clusters in end stage osteoarthritis since the primary cells used were derived from a patient undergoing total knee replacement surgery at end stage osteoarthritis. (ii) How does mechanical compression affect ECM production by chondrosarcoma cells either cultured as micromasses or single cells? To that end, we adapted a previously developed mechanical actuation unit integrated in a cartilage-on-chip platform to apply compressive forces on cell-laden hydrogels in a cyclic manner.^19^ This mechanical actuation unit comprises a thin membrane which is deformed in a fully programmable manner by applying pressure in one large chamber. The device also includes a perfusion channel to provide nutrients to cells. We adopted a simplified version of our model to increase the throughput of the system and increase the number of conditions studied. We considered four cellular models: CH2879 chondrosarcoma cells or one primary OA chondrocyte either cultured as single cells or as micromasses of approximately *±* 50 cells in an agarose hydrogel. Micromasses of primary chondrocytes morphologically resemble chondrocyte clusters as found in third- and fourth-stage OA.^20^ Alternatively, chondrocytes clustering is routinely used to boost cartilage matrix production in in vitro cell culture.^4^ The organ on chip cultures were either stimulated with physiological load in the form of cyclic compression or left untreated as a static control. A physiological stimulus was used since also cell aggregates in OA experience physiological compression. Moreover, literature on the impact of mechanical loading on chondrosarcoma is scarce; therefore, we compared the response of primary chondrocytes with chondrosarcoma. Specifically, we examined the impact of cyclic compression on cell/micromass deformation, the gene expression levels for well-established chondrocyte markers and the production of ECM proteins. Overall, our data reveal that the two cell types respond differently at the gene and protein level when exposed to the same mechanical stimuli, irrespective of culturing method as single cells or as micromasses.

## 2. Star Methods

### 2.1. Cell Culture

Primary chondrocytes which were isolated from a healthy looking region of articular cartilage (female donor, age 60 years, isolated from the knee, end stage OA grade 4) were derived from a patient undergoing a knee arthroplasty and mCherry-CH2879 chondrosarcoma cells^21^ were cultured in T175 culture flasks (Cellstar®, Greiner bio-one, Kremsmünster, Austria) in standard chondrocyte proliferation medium (DMEM (Dulbecco’s Modified Eagle Medium) supplemented with 10% FBS (Fetal Bovine Serum), 0.2 mM ascorbic acid 2-phosphate, 0.1 mM non-essential amino acids, 100 U/mL penicillin, 100 µg/mL streptomycin, and 4 mM proline). Approval for isolation and use of primary chondrocytes was obtained from a local medical ethical committee. Medium was refreshed every 3 days. For passaging, cells were washed with PBS and afterwards, a trypsin-EDTA (1X) solution (0.25%/0.1 mM, Invitrogen, Thermo Fisher Scientific, Carlsbad, CA, USA) was added. Cells were seeded in a new T175 flask at a concentration of 500,000 cells/flask. Human chondrocytes were used at passage 4 and the chondrosarcoma cells at passages 8-10.

### 2.2. Generation of Clonal Luciferase Expressing Chondrosarcoma Cell Lines mCherry-CH2879

The lentiviral vector pLV.CMV.mCherry.bc.PURO (kind gift from Eric Kaijzel, LUMC) and packaging vectors pMD 2/VSV-G, pMD L/pRRE, and pRSV-rev were transfected into Hek293t cell to produce luciferase lentiviral particles. The chondrosarcoma cell line CH2879 was cultured in RPMI 1640 medium (Invitrogen) supplemented with 10% (v/v) heat-inactivated foetal bovine serum (Lonza), 1% glutaMAX (Invitrogen), and 50 μg/mL penicillin/streptomycin (MP Biomedicals). After infection with luciferase lentiviral particles, transduced cells were selected using 2 μg/mL puromycin. Following antibiotic selection, single-cell-derived clonals were obtained using limited dilution and screened for mCherry expression.

### 2.3. OoC Device Fabrication

The organ-on-chip devices were produced as previously described by soft-lithography, using polydimethylsiloxane (PDMS) and a SU-8-on-silicon mold.^19^ Briefly, SU-8 100 photoresist (MicroChem, Westborough, MA, USA) was spin-coated on a silicon wafer to a final thickness of 250 µm, next exposed to UV-light through a quartz mask and developed according to the manufacturer protocol. A mixture of PDMS pre-polymer and curing agent (Sylgard 184, Dow Corning, Midland, MI, USA) with a weight ratio of 20:1 was poured on the mold, degassed, and placed in an oven at 60°C for 24 h. Inlets and outlets were created with 1-mm and 2-mm punchers, respectively. Slabs of PDMS with a 4-mm thickness were produced by pouring the same 20:1 PDMS mixture in a petri dish and curing it under the same conditions. The 4-mm PDMS slabs and microscopy glass slides were plasma-treated (Cute, Femto Science, South Korea) and bonded together, before being assembled to the PDMS fluidic layer, which was also exposed to plasma. Before use, OoC devices were placed again in an oven at 60°C for 24 h.

### 2.4. OoC Device Characterization

The PDMS membrane deformation was first characterized in four devices by applying homogenous positive pressure, as previously reported.^19^ The deformation was obtained by subtracting the membrane initial position to the one recorded while applying compressive pressure (Figure S14). A pressure controller (Flow EZ, 2,000 mbar, Fluigent, Paris, France) and associated automation software (Fluigent) was used to apply well-defined pressure levels in the OoC device. Next, the hydrogel deformation in the device was quantified by evaluating the displacement of 15-µm diameter polystyrene microbeads (Kisker, Burgsteinfurt, Germany) added at a concentration of 60 µg/mL in low-melting agarose (low melting temperature agarose (LMT), Invitrogen). Next, 100 µL of food dye (JO-LA, Frambozen, Lambrecht, Germany) was diluted 10 times in PBS and injected manually in the perfusion channel to evaluate diffusion into the agarose hydrogel upon deflection of the membrane (300 mbar applied pressure at 1 Hz). Similarly, 10 µL of Rhodamine B (100 mg/mL) (Sigma-Aldrich, St. Louis, Missouri, USA) was diluted in 1 mL of PBS with the same mechanical stimulation protocol. To quantify diffusion, nine independent zones were considered in the samples, three close to the pillars, three in the middle and three close to the actuation unit. The gray intensity in each zone was evaluated as a function of time using ImageJ.^22^

### 2.5. Micromass Formation

Micromasses of both cell types were formed using an agarose microwell array,^23^ each microwell containing in average 50 cells (Figure S1). The microwell array was created by pouring a 3% w/v agarose solution (UltraPure™ Agarose, Thermo Fisher Scientific) onto a PDMS mold and waiting for agarose gelation. Each microwell array, which contained 3600 wells of 200-µm diameter and 200-µm depth, was placed in a 12-well plate. Approximately 300,000 cells were seeded in each microwell array, after which the well plate was centrifuged for 3 min at 300 rpm. After centrifugation, 1 mL of standard chondrocyte proliferation medium was added. Cells clustered within one day to yield micromasses, that were collected and seeded in the OoC devices on day 2. For this, the agarose microwell array containing the micromasses was flipped and centrifuged for 1 min at 500 rpm, which allowed recovering 60-70% of the micromasses. After discarding of the agarose microwell array, micromasses were kept in medium before seeding in the OoC device.

### 2.6. Cell Size Measurements Using Flow Cytometry

The size of the chondrocytes and chondrosarcoma cells was evaluated using flow cytometry (Becton Dickinson (BD), FACS Aria II, BD Biosciences, Becton, NJ, USA). Specifically, 1,000,000 cells were resuspended in DMEM without any supplement and kept on ice. Cell size was compared to a range (2.0-16.4 µm) of polystyrene micro-beads (Spherotech, Lake Forest, IL, USA).

### 2.7. Preparation of the OoC Devices With Chondrocytes and Chondrosarcoma Cells

As already mentioned, four different cellular models were considered in this work: single cell suspensions in agarose of primary human chondrocytes or chondrosarcoma cells, and micromasses of the same cell types. Based on previous work,^19^ 2% (w/v) agarose (LMT agarose, Invitrogen) was used as a hydrogel matrix to seed single cells or micromasses in the OoC device. A concentration of 1,500,000 cells per mL (single cell suspension) or pools of two agarose microwell arrays per 2 mL, resuspended in hydrogel, and seeded in the platform. Standard chondrocyte proliferation medium was added to the perfusion channel in the OoC device. Medium was changed daily by pipetting.

### 2.8. Cell Deformation Upon Compression

All four cellular models in agarose hydrogel were stimulated with compression at 0 (static), 100 mbar, 200 mbar, 300 mbar, 500 mbar and 700 mbar at day 1 in the OoC device, the pressure being progressively increased in the actuation chamber. Pictures were taken at each compression level. Three sections were considered (zone width of 420 µm) in the hydrogel chamber: close to the membrane (top), in the middle and close to the pillars (bottom). Pictures were subsequently analyzed with ImageJ. In each zone, the cell deformation was quantified along the axis of stimulation for ten different cells or micromasses, by comparing the cell dimension at rest (0 mbar) and under compression for the different applied pressures, using the following formula:

$$c=\frac{\Delta L}{L}=\left(\frac{L-l}{L}\right)*100$$

Where c is the projected cell/micromass deformation (%), L the initial dimension of the cell at rest (0 mbar) and l its dimension under compression (100, 200, 300, 500 or 700 mbar), both along the axis of stimulation.

### 2.9. Gene Expression Analysis

OoC devices were cultured either under static conditions or with compressive forces for four days (stimulation 1 hour a day at 1 Hz, using 300 mbar pressure) starting from day 1. Three hours after the last stimulation cycle, the OoC devices were disassembled, the cell-laden agarose hydrogels retrieved and placed in the RNA extraction buffer (RNeasy Micro Kit (cat. number 74004, QIAGEN, Hilden, Germany), by pooling three independent devices to obtain sufficient RNA. Next, iScript™ cDNA Synthesis Kit (Bio-Rad, Hercules, CA, USA) was used to synthesize cDNA. At last, qPCR was performed on the resulting cDNA using SensiMix™ SYBR® and Fluorescein Kit (Bioline, London, UK) on a CFX Connect Real-Time System (Bio-Rad). Gene expression levels were determined for *GAPDH* (S17)*, COL1A1, SOX9, ACAN, MMP13* (See Table S1 for primer sequences). The values obtained were normalized with *GAPDH* expression level and the 2^^(-ΔCt)^ used to determine the differences between conditions. The *GAPDH* expression was found to be stable for all the samples (data shown in Figure S17).

### 2.10. Immunofluorescence and Extracellular Matrix Production

To determine the effect of prolonged mechanical stimulation on the ECM production, culture was extended to 10 days with compressive forces as before (300 mbar at 1 Hz for 1 hour a day, with rest on day 5 and day 10) or not. Samples were sacrificed as before by disassembling the OoC device, washed with PBS, fixed in 4% buffered formaldehyde solution, washed twice with PBS, permeabilized with 0.25% Triton X-100 in PBS for 30 min, washed again with PBS and blocked with 1% Bovine Serum Albumin in PBS for 1 h (all at room temperature). Next, the primary antibody was added overnight at 4^o^C following a dilution of 1:100. After washing with PBS three times, the secondary antibody was added for 1 h followed by washing. Lastly, DAPI (4′,6-diamidino-2-phenylindole, Thermo Fisher Scientific) was added for 15 min at room temperature, before washing three times with PBS. Primary antibodies were ACAN (Anti-ACAN antibody [6-B-4] (ab3778), Abcam, Cambridge, UK), COL1 (Collagen I Antibody (NB600-408), Novus biological Abingdon, UK), COL2 (COL2A1 Antibody (ab34712), Abcam) and COL6 (Anti-Collagen VI antibody (ab182744), Abcam). Secondary antibodies were Alexa fluor 647 anti-mouse (ab150107, Abcam) and Alexa fluor 488 anti-rabbit (A-11008, Invitrogen), all used with a dilution of 1:1000. Images were taken with a NIKON Eclipse TI confocal microscope (NIKON, Tokyo, Japan). Immunofluorescence images were analyzed by framing the micromasses in a square and using the plot profile function of ImageJ (Figure S15). Here ImageJ gives the average of the points present in the x axis (Figure S15). The average of each point in the x axis is then plotted as histogram. The average of 5-8 histograms, each belonging to one micromass, was used to create the histogram presented. To determine the presence of a protein core in the histogram, a single line cutting in half the micromass was used (Figure S16). For “core” assessment, a line was drawn through the geometric center of the micromass. A “core” was defined as a central fluorescence maximum occupying the inner region of the aggregate; a “peripheral shell/crown” was defined as fluorescence enriched near the aggregate boundary; and “protrusions” were defined as discrete peripheral extensions producing local intensity peaks outside the main aggregate contour.

### 2.11. Quantification of Spatial ECM Organization

Spatial distribution of ECM-associated fluorescence around micromasses was evaluated using Fiji/ImageJ. Individual micromasses were first identified in maximum-intensity projections/representative optical sections and manually outlined as regions of interest (ROIs). For each micromass, the geometric center and the boundary contour were determined from the ROI. Fluorescence intensity profiles were then extracted along a predefined axis corresponding to the loading direction and, where indicated, also along the orthogonal axis. To enable comparison between micromasses of different size, distances were normalized from 0 to 1, where 0 corresponded to the side facing the actuation membrane and 1 to the opposite side. Note that the hydrogels presented on one side a flat surface (the one in front of the mechanical stimulation) while the opposite site presented with dents due to the pillar arrangement. This geometrical feature of the chip was used to orient cells relative to the direction of load.

To reduce subjective interpretation, spatial patterns were classified using predefined operational criteria. A **core-like distribution** was defined as a fluorescence maximum located within the central region of the micromass (for example, the central 30–40% of the normalized diameter). A **crown/shell-like distribution** was defined as fluorescence enrichment predominantly located at the micromass periphery, with lower relative intensity in the center. **Protrusions** were defined as discrete local extensions of fluorescence signal beyond the main rounded micromass contour, producing localized peripheral peaks in the intensity profile and visible as non-uniform outward extensions in the corresponding image. These descriptors were used as semiquantitative pattern categories and not as absolute morphometric classes.

For each condition, multiple independent micromasses were analyzed, and intensity profiles were plotted individually and/or averaged per condition. The number of micromasses analyzed per group is now indicated in the figure legend/supplementary methods.

### 2.12. Statistical Analysis

Statistical analyses were performed in Origin. Inferential statistical testing was applied only to datasets for which clearly defined biological replicates and directly comparable quantitative readouts were available, such as the gene-expression data. For comparisons among multiple groups, one-way ANOVA followed by Tukey’s post-hoc test was applied. The assumptions underlying ANOVA were independence of observations, approximate normality of the data/residuals, and homogeneity of variance across groups. Independence was defined at the level of biological replicates. Approximate normality and variance homogeneity were assessed by visual inspection of the data distribution and spread across groups. To improve transparency, individual data points are shown where possible.

By contrast, representative microscopy images, deformation maps, and semiquantitative analyses of ECM spatial organization were included primarily to illustrate structural organization and comparative trends across conditions. These datasets were therefore interpreted descriptively or semiquantitatively and were not subjected to formal inferential testing unless explicitly indicated. This distinction is clarified in the corresponding figure legends and Results sections. A *p* value < 0.05 was considered statistically significant.

## 3. Results and Discussion

### 3.1. Design of the OoC Device

**Figure 1.**
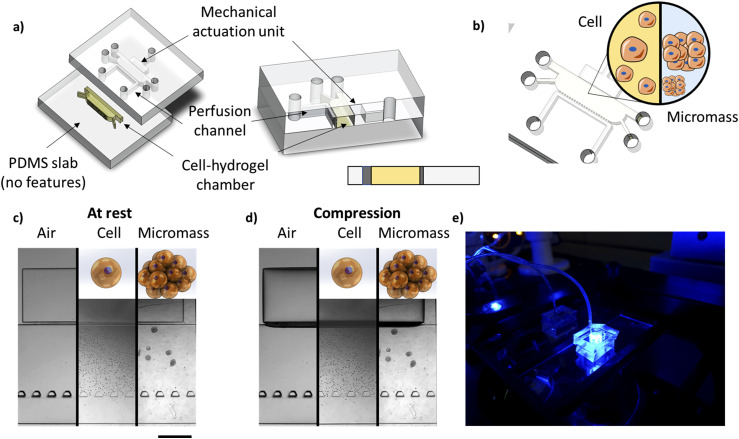
OoC device equipped with a mechanical actuation unit, for the culture of chondrocytes and chondrosarcoma cells in hydrogel, as single cells or micromasses, and their on-demand exposure to (cyclic) compressive forces. (A) Schematic overview of the PDMS OoC device containing one actuation chamber, a hydrogel section, and a perfusion channel. (B) Overview of the four cellular models; single-cell suspensions and micromasses of primary chondrocytes or chondrosarcoma cells. (C) Top mosaic picture of the OoC device with, from left to right, hydrogel only a single cell suspension or micromasses at rest. (D) same as in (C) under application of compressive forces (300 mbar, here). Scale bar: 500 µm. (E) OoC device connected to the external actuation system placed on a fluorescence microscope

The OoC device is reminiscent of a device we previously published to apply mechanical stimulation on cell-laden hydrogels, with one main adaptation (Figure 1A, B and E).^19^ The device consists of one actuation unit, a cell-hydrogel section, and a perfusion channel instead of 3 actuation units. The PDMS membrane is 50-µm thick and 250-µm high. The device was produced using a standard soft-lithography procedure, using a SU-8-on-silicon mold and a 20:1 (monomer:cross-linker) PDMS mixture to maximize membrane deflection while ensuring maintenance of its structural integrity.^19^ Furthermore, the resulting PDMS structures were bonded to a glass substrate coated with a 4-mm thick PDMS layer, not presenting features, also in the view of enhancing membrane deformation.^19^ Compared to our previous publications^19,24^, one single actuation chamber was employed to exert compressive load only. The device lacked bulk shear forces (Figure 1C and D) which would require three independently operational pressure chambers^19,24^.

A key novelty of the present work is not the use of compression on chondrocytes per se, but the combination of four features within one controlled organ-on-chip framework: (i) direct comparison of single cells and micromasses, (ii) inclusion of both primary OA chondrocytes and chondrosarcoma cells, (iii) coupling of acute deformation measurements with later gene- and ECM-level readouts, and (iv) assessment of semi-quantitative spatial ECM organization relative to the loading direction. To our knowledge, this combination has not previously been reported for cartilage-derived benign and malignant cell models under the same cyclic compression regime. The conceptual contribution of this study is therefore the demonstration that, within one defined OoC setting, cellular organization can modify compression-associated phenotypes and matrix arrangement.

### 3.2. Micromass Production and Characterization

Micromasses were successfully produced for both chondrocytes isolated from healthy looking regions of articular cartilage derived from osteoartritic patients undergoing knee arthroplasty and the chondrosarcoma cell line CH2879 stably transduced with an mCherry reporter construct^21^) using an in-house-developed procedure using an agarose microwell array^23^ (Figure S1). Specifically, 300,000 cells were seeded in a microwell array comprising 3,600 microwells (200-µm diameter and 200-µm depth). Micromasses, which contained on average 50 cells each (Figure S1), remained stable even after the on-chip injection procedure, which confirms that cell-cell interactions were sufficient to keep micromasses intact after injection in the OoC device. We characterized the micromass size over time through imaging in the microwell array. For the chondrosarcoma cells, the micromass diameter increased from 106.9 ± 8.7 µm (day 1) to 110.9 ± 12.0 µm (day 2) (n = 26 micromasses) and reached 159.5 ± 9.4 µm on day 6 (Figure S2). Although the same seeding density was employed for both cell types in the microwell arrays, the average size of chondrocyte micromasses (n = 26) was smaller, and a partial decrease was detected at day 6 (69.7 ± 4.2 µm on day 1 *vs*. 59.7 ± 9.6 µm on day 6) (Figure S2). This decrease in size could be accounted for the fact that cells experience a new three-dimensional environment that is likely to impact their cytoskeleton. This phenomenon known as compaction, is accompanied by a decrease in cellular volume.^25^ This discrepancy in behavior between the two cell types could be related to the hyper-proliferative nature of the chondrosarcoma cells, as previously reported by Leva et al.^26^ Finally, cells remained alive within the micromasses and no necrotic or hypoxic core was observed since the micromass size was smaller than 200-300 µm.^27^

### 3.3. OoC Platform Characterization: Membrane Deflection, Hydrogel Deformation and Nutrient Delivery

We characterized the OoC platform to evaluate its performance in terms of membrane deflection as a function of the applied pressure (compressive forces only, from 0 to 700 mbar, (Figure S3). Pressure was applied stepwise in the actuation chamber using a pressure controller (Flow EZ, 2,000 mbar, Fluigent) and associated automation software (Fluigent). All reported values refer to the pressure applied via the pressure controller. As expected, and in line with our previous work yet using a slightly different design,^19^ the higher the applied pressure (from 0 to 700 mbar), the greater the deflection of the membrane (Video S1). Specifically, at a low applied pressure (100 mbar), the displacement was 26.5 ± 5.7 µm *vs*. 195.3 ± 22.8 µm for a pressure 700 mbar (Figure S3 a,b). Next, the deformation of LMT (low-melting temperature agarose, 2% w/v) agarose hydrogel was quantified upon application of a pressure. For this, the agarose was supplemented with polystyrene microbeads (15 µm in size, 60 µg/mL) and their displacement was assessed when exposed to the same pressure range. The cell-hydrogel chamber was divided in six regions of each 210-µm in width starting from the membrane (R0-violet) to the pillars (R5-orange), and the displacement of eight microbeads per region was quantified (Figure S3c). This division in six zones provides a proper representation of the hydrogel deformation within the entire hydrogel chamber, which is 1260 µm in width. For each applied pressure, the agarose deformation in R0 was greater, resulting in a larger microbead displacement (approx. 200 µm at an applied pressure of 700 mbar) compared to R5 (approx. 30 µm at the same pressure) (Figure S3 d). Next, the efficiency to deliver nutrients to the entire construct was evaluated using a food dye (JO-LA, diluted 10 x in PBS) and rhodamine B pipetted in the perfusion channel (Video S2 & Figure S4). Upon application of compressive forces (300 mbar at a 1-Hz frequency), the food dye and the rhodamine B solution readily diffused within seconds from its injection in the perfusion channel through the entire construct (Figure S3 e,f), which can be explained, on one hand, by the porous structure of the agarose hydrogel, and, on the other hand, by the presence of a possible gap between the agarose and the PDMS since the hydrogel was not covalently attached to the PDMS, allowing thereby medium “flow” above and below the agarose matrix. This characterization altogether demonstrates that nutrients can efficiently reach cells in the hydrogel in the OoC device in both static and dynamic (*e.g*., with compression) culture conditions. Finally, chondrocyte deformation was quantified in the agarose hydrogel construct as a function of the applied pressure; it roughly increased with the applied pressure, which is discussed in more detail in the following sections for all four cellular models (Figure S3 g,h).

### 3.4. Mechanical Stimulation Differently Impacts Deformation of Chondrocytes and Chondrosarcoma Cells Cultured Either as Single Cells or Micromasses

**Figure 2.**
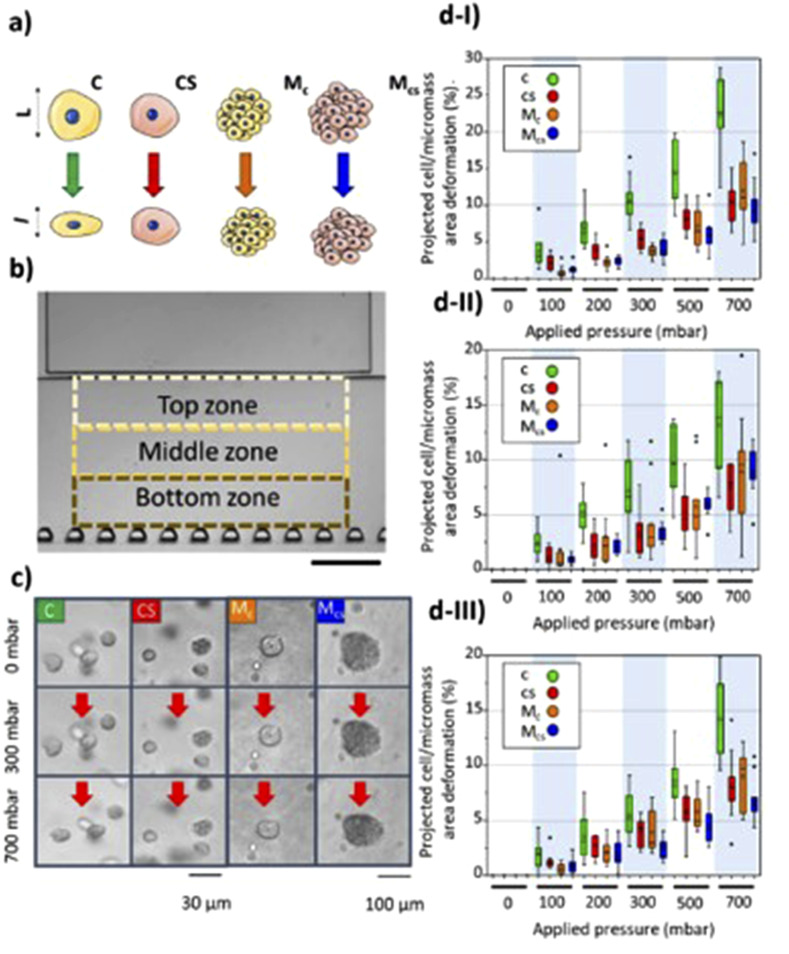
Deformation of chondrocytes and chondrosarcoma cells, cultured either as single cells or micromasses upon application of compressive forces. The four cellular models are: c: single cell chondrocytes (green), cs: single chondrosarcoma cells (red), M_c_: chondrocyte micromasses (orange), M_cs_: chondrosarcoma cell micromasses (blue). (A) Schematic overview of the cell and micromass deformation upon application of compression, showing the direction along which cell deformation is evaluated, which is the same as the axis along which compressive forces are applied. (B) Top view of an empty OoC device depicting the three zones considered for the cell/micromass deformation analysis: top close to the actuation unit, middle and bottom close to perfusion channel (all 420 µm in width). Scale bar: 500 µm. (C) Single cell and micromass deformation for both cell types at rest (0 mbar) and under application of compression at 300 and 700 mbar. Red arrows indicate the direction of the forces applied. (D) I-III) Graphs representing the average deformation of ten individual cells or micromasses in the top (I), middle (II), and bottom (III) zones, as defined in (B). Black dots indicate outliers. Note the differences in the y-axis scale between (I) and (II) and (III)

The deformation of all four cellular models (Figure 2A) was evaluated in the OoC platform for the same pressure range as before (0-700 mbar) and quantified in three different zones of equal width: the top zone (close to the membrane), the middle, and the bottom zone (close to the pillars) (Figure 2A). For this, a suspension of single chondrocytes (one donor) or chondrosarcoma cells at a concentration of 1,500,00 million cells/ml or the recovered content of two agarose microwell arrays was resuspended in LMT agarose hydrogel (2% w/v) and injected in the device. Overall, the cell and micromass deformation increased with the applied pressure which depended on their position in the hydrogel construct with respect to the membrane (along the stimulation axis) (Figure 2). For instance, the deformation of individual chondrocytes in the top zone was 3.7 ± 2.3% at 100 mbar *vs*. 22.3 ± 5.1% at 700 mbar, and was much lower in the bottom zone (1.9 ± 1.3% at 100 mbar *vs*. 14.3 ± 3.5% at 700 mbar). Interestingly, significant differences were found between chondrocytes and the three other cellular models. Chondrosarcoma single cell deformation was lower than that of single chondrocytes (respectively, 2.2 ± 1.0% *vs*. 3.7 ± 2.3% at 100 mbar and 10.4 ± 2.6% *vs*. 22.3 ± 5.1% at 700 mbar, both in the top zone), which may be related to differences in size and stiffness of these two cell types. First, the overall volume of chondrocytes was larger than that of chondrosarcoma cells, as evaluated by flow cytometry (Figure S5). Next, the reported Young’s modulus of chondrosarcoma cells, although for a different cell line than used in this study, is higher than that of chondrocytes (1.27 ± 0.86 kPa for the FS090 chondrosarcoma cells^28^ against approximately 0.6 kPa for primary chondrocytes.^29^

**Figure 3.**
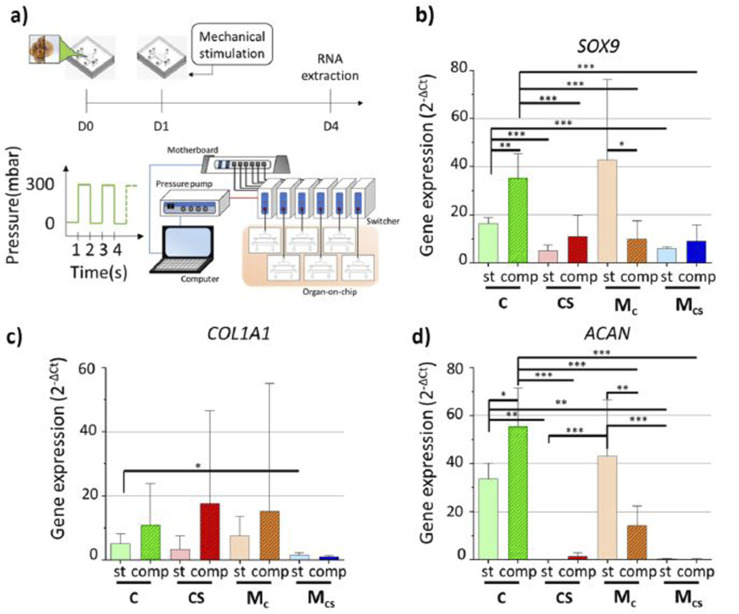
Effect of compressive forces on gene expression levels in chondrocytes and chondrosarcoma cells cultured as single cells or micromasses in the OoC platform. (A) Schematic representation of the experimental design. Mechanical stimulation is applied from day 1 to day 4. Gene expression levels of *SOX9* (B), *COL1A1* (C) and *ACAN* (D). Four cellular models are considered: c: single chondrocytes (green), cs: single chondrosarcoma cells (red), M_c_: chondrocyte micromasses (orange), M_cs_: chondrosarcoma cell micromasses (blue). Each condition consists of nine organ-on-chip devices and three independent samples. P value: 0.1>Pvalue>0.05 *, 0.05>Pvalue>0.01 **, 0.01>Pvalue>0.001 ***

Remarkably, the trend in compression-induced deformation of the single chondrosarcoma cells is, to a large extent, comparable to that of chondrocytes or chondrosarcoma cells cultured as micromasses. Of particular interest is the behavior of chondrocyte micromasses, which proved to be more resistant to compression in terms of cell deformation compared to their single cell counterparts. This observation opens the intriguing thought that cell clusters, as observed in late stage of OA cartilage, could be an adaptive response to increase their resistance to load. In fact, cell load is expected to be much higher in dysfunctional OA cartilage compared to normal cartilage due to the degradation of the superficial layers.^30^ Micromass deformation was similar for chondrocytes and chondrosarcoma cells in each of the three zones in the hydrogel chamber with no detectable difference (for chondrocytes and chondrosarcoma micromasses, respectively, 0.9 ± 0.8 *vs*. 1.3 ± 0.7 at 100 mbar; 11.9 ± 4.2 *vs.* 9.6 ± 3.5 at 700 mbar – in the zone closest to the mechanical actuation unit (top zone) (Figure 3D-I); and 0.6 ± 0.5 *vs*. 0.9 ± 0.6 at 100 mbar and 9.0 ± 2.6 *vs*. 6.8 ± 2.1 at 700 mbar in the zone closest to the perfusion channel (bottom zone) (Figure 3D-III)). Our results show that chondrosarcoma cells either cultured as single cells or micromasses are more resistant to compressive forces than chondrocytes cultured as single cells in terms of cell deformation, and clustering of chondrocytes into micromasses modifies their response to these compressive forces. It should be noted that LMT agarose does limit cell attachment and thus integrin-mediated mechanotransduction of the cells, which can affect ECM and PCM organization, as well as load distribution. Also, the alterned poroelasticity might have an effect on transport and mechanical cues. However, we compared single chondrocytes vs single chondrosarcoma cells and chondrocyte micromass vs chondrosarcoma micromass embedded in the same agarose matrix enabling the direct comparison between the various conditions.^31^

Importantly, these results should be interpreted in the context of the specific mechanical environment created by the present platform. Under the defined conditions of this study, including the use of one OA donor, one chondrosarcoma cell line, a 2% LMT agarose matrix, the present chip geometry, and a cyclic compression protocol of 300 mbar at 1 Hz, cellular organization was associated with differences in compression response. However, the extent to which these differences reflect intrinsic effects of single-cell versus micromass organization, as opposed to matrix-dependent differences in load transmission, cannot yet be separated in the current design. Because agarose stiffness, poroelasticity, and lack of a fibrillar adhesive network influence how mechanical forces are distributed to embedded cells, the biological response likely reflects both cell organization and matrix-mediated force transmission.

From a cartilage mechanobiology perspective, an important next step will be to repeat the study across multiple agarose stiffnesses or matrix systems, as this would help disentangle organization-dependent effects from matrix-dependent effects on mechanical load transfer, deformation, and downstream ECM production.

### 3.5. Gene and Protein Expression Differs Between Single Cells and Micromasses of Chondrocytes and Chondrosarcoma Cells Cultured Under Static Conditions or Exposed to Cyclic Compression

Both chondrocytes and chondrosarcoma cells are known to produce high levels of ECM proteins. While mechanical stimulation is a driving factor for the ECM production in primary chondrocytes,^32^ its role in matrix production in chondrosarcoma tissues is less clear. In contrast to chondrocyte micromasses, little is known on how ECM expression is affected when chondrosarcoma cells are cultured as micromasses. Current studies focus on the critical regulators of the progression of chondrosarcomas^33,34^ and approaches to target this type of tumor.^35,36^ Moreover, to the best of our knowledge, no research has so far investigated the effect of active mechanical stimulation on micromasses of chondrosarcoma cells and little is known about the behavior of chondrocyte clusters *in vitro*. We therefore looked at the impact of mechanical stimulation in the form of compressive forces in each of the 4 culture conditions, on the mRNA expression of *SOX9, ACAN, COL1A1* and *MMP-13*. Additionally, we studied the expression of ACAN, COL1A1, ACAN and COL2A1 at the protein level. As depicted in Figure 3A, after loading in the OoC platform cells were allowed to recover for one day before cyclic compression (300 mbar, at 1 Hz for 1 hour per day) was applied for 3 days in a row. At the end of the experiments, the device was disassembled, the cell-hydrogel construct was recovered for RNA extraction or fixed for immunofluorescence staining. For these experiments, three independent samples were considered per condition.

The transcription factor *SOX9* is highly expressed in chondrocytes and plays a role in multiple pathways like the production of ECM proteins, or the activation of other transcription factors.^37^ In chondrosarcoma cells, it has been shown that downregulation of *SOX9* results in dedifferentiation which is associated with an increase in cancer aggressiveness.^38^ In this study, *SOX9* was upregulated (0.05<P_v_<0.001) in a single OA chondrocyte cultured as a single cell suspension and exposed to compressive forces, compared to static culture conditions (Figure 3B), which is in line with recent literature.^39^ Still, depending on the exact model used, the length of the mechanical stimulation period and the chondrocyte state (disease *vs*. healthy) this increase in *SOX9* is not always detected.^40^ *SOX9* upregulation upon mechanical stimulation was less pronounced for the CH2879 chondrosarcoma cells cultured as single cells in the hydrogel construct. Culturing chondrocytes as micromasses in static conditions already resulted in an increased expression of *SOX9*, with similar expression level to that of single chondrocytes exposed to mechanical stimulation. Interestingly, application of cyclic compressive forces on chondrocyte micromasses led to an opposite effect, with a drastic reduction in *SOX9* mRNA expression, below that observed for single chondrocytes in static culture in a 3D hydrogel matrix.^37^ Also, in single donor chondrocyte clusters found in end-stage OA *SOX9* mRNA expression is reduced and (hyper)physiological mechanical cues might be responsible for this downregulation.^37^ Taken together, we speculate that clustering of this single donor OA chondrocyte enables them to better deal with mechanical stress, but that this enhanced resistance comes at the expense of a loss in *SOX9* mRNA expression which could potentially lead to loss of the cartilage phenotype and/or hypertrophic differentiation.^41^ Additional hypertrophic and osteogenic markers, such as COL10A1, RUNX2, and OPN, would be required to test this possibility directly in future studies. In contrast, CH2879 chondrosarcoma single cells and micromasses showed a different SOX9 expression pattern under the tested loading regime, with similar expression between the two culture formats and a slight upregulation upon mechanical stimulation.

Next, we evaluated the expression of *COL1A1* at the gene and protein level. COL1A1 is an ECM protein which is present at low levels in the pericellular matrix of healthy chondrocytes but expressed at considerably higher levels in diseased cartilage.^42^ Still, little is known on *COL1A1* expression by chondrosarcomas.^26^ In this study, exposure to compressive forces resulted in an upregulation of *COL1A1* expression in all conditions apart from chondrosarcoma micromasses, for which the expression did not change compared to static culture (Figure 3C). However, mechanical stimulation of chondrocyte micromasses reduced the collagen I protein expression at day 4 in respect to the static conditions (Figure S6). On the contrary, collagen I was upregulated in mechanically stimulated conditions at day 4 in chondrosarcoma micromasses (Figure S7). This suggests a difference in cellular response of the two cell types towards mechanical stimuli.

*ACAN* mRNA, the gene encoding the cartilage-specific proteoglycan, was mainly expressed in chondrocytes cultured either as single cells or micromasses, but at a much lower level in chondrosarcoma cells (both single cells and micromasses, with or without exposure to compression) (Figure 3D). Interestingly, the trend in *ACAN* expression level for chondrocytes followed the expression of *SOX9* with an upregulation in single cells and a downregulation in micromasses upon application of compressive forces. The similar trend in regulation of these two markers is in line with previous literature as SOX9 is known to be upregulating ACAN.^37^ Interestingly, the protein level of aggrecan remained similar or was reduced at day 4 both for chondrocyte and chondrosarcoma micromasses (Figure S6 & S7). Collagen II, one of the typical cartilage markers, was increased upon mechanical stimuli in chondrocyte micromasses while for chondrosarcoma it mostly reduced (Figure S6 and S7). No clear differences were observed for chondrocytes and chondrosarcoma cultured as single cells in collagen I, aggrecan and collagen II expression (Figure S8 & S9).

The expression levels of *MMP-13*, a member of the matrix metalloproteinase (MMP) family increased in both single cell models upon application of compression, as already reported for chondrocytes,^43^ while it decreased in chondrocyte micromasses (Figure S9). Altogether, these results reveal differences in behavior between the two cell types but also whether cells were cultured as single cells or as micromasses. We noted however that gene expression levels did not always correlate with the protein expression and does not provide any information on their spatial organization in (and around) the cells and micromasses. Therefore, as a next step, we examined the production of proteins using immunostaining during longer periods of cell culture.

### 3.6. Exposure to Compressive Forces Enhances ECM Production in Both Chondrocytes and Chondrosarcoma Cells

**Figure 4.**
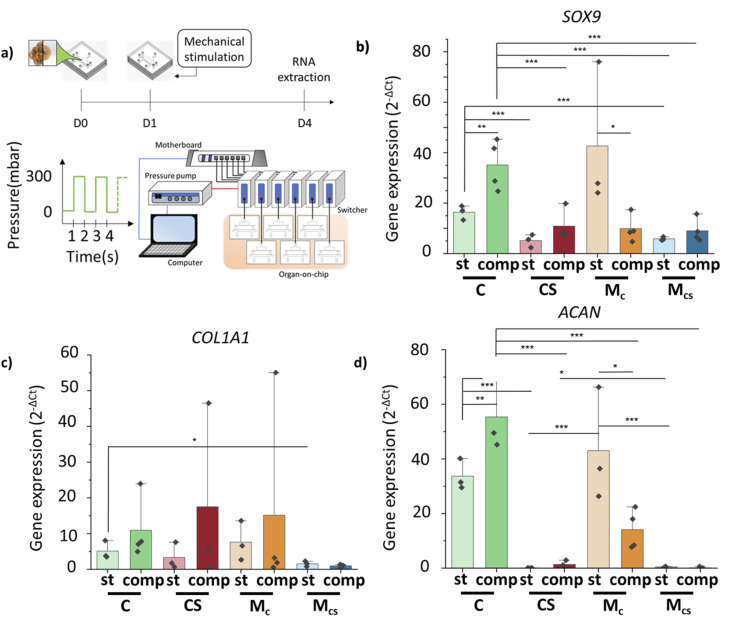
ECM production by chondrocytes and chondrosarcoma cells cultured as single cells under static and compressive conditions. (A) Schematic summary of the experimental design. Chondrocytes expressing ACAN (red) and COL1A1 (green) (B) or COL1A1, 2, and 6 (green) (C) in static conditions (top) and exposed to cyclic compression (bottom). Chondrosarcoma expressing ACAN (red) and COL1A1 (green) (D) or COL1A1, 2, 6 (green) (E) in static culture (top) and exposed to compression (bottom). White arrows indicate protein deposition in the surrounding of the cells which we consider as protein protrusions. Percentage of cells presenting protein protrusions out of the 40 considered cells for chondrocytes (F) and chondrosarcoma cells (G). Scale bars: 20 µm

Chondrocytes within the cartilage reside in the pericellular matrix, a shell which functions as a protective layer and acts as a signal transducer. Chondrosarcoma cells, on the contrary, reside in an ossified matrix in which, thanks to the porosity of this environment, they can easily proliferate and expand. Although being exposed to different conditions, both cell types are known to produce the same types of ECM proteins. Hence, we investigated whether the behavior of these two cell types was similar in terms of ACAN (Aggrecan), COL1 (COL1A1 I), COL2A1 and COL6 (COL6A1 VI) production at the protein level (Figure 4). For this, the cellular models were kept in culture in the device for 10 days, and were either exposed to cyclic compression or not. Evaluation was performed at day 1, 4 and 10 of culture. As in the previous section, cyclic compression (300 mbar, 1 Hz) was applied for one hour a day, after one day of culture in the platform and with a break on days 4 and 5 (Figure 4A). The addition of two rest days were given to allow the cells to respond to the stimulus both by producing ECM and adjusting their cytoskeleton.^40,44^ After this period of 1, 4 and 10 days, the cell-laden hydrogels were retrieved from the platform, fixed with 4% formaldehyde, immune-stained for the above-listed markers, counter-stained with DAPI (nuclear dye) and imaged by confocal microscopy. Day 1 and day 4 were used to determine possible initial differences between the cultures. As all the cells presented proteins, such as collagen I and II, in proximity of their membrane, in this study we considered the proteins produced in the surrounding matrix (protrusions) to identify differences between conditions (Figure 4B). Since cells stained positive for COL1A1 and COL2A1 in proximity of the cell membrane, we considered protein expression in the surrounding matrix to identify differences between conditions (Figure 4B). The presence of those proteins around the single cells was determined for 40 cells in total at different positions in the chamber, as highlighted by white arrows (Figure 4A-D) at day 10. No early time points (day 1 and 4) were considered as no clear differences could be observed (Figure S8 & S9). For both chondrocytes and chondrosarcoma cells a slight upregulation was detected for ACAN when applying compressive forces, in terms of % single cells expressing this marker (35% chondrocytes and 25% chondrosarcoma cells) compared to static conditions (25% chondrocyte & 19% chondrosarcoma). A similar but more pronounced trend was found for COL1A1 (86% chondrocytes and 56% chondrosarcoma cells in compressive culture *vs*. 18% chondrocytes and 37% chondrosarcoma cells in static culture) (Figure 4E and F). COL2A1 and COL6A1 gave similar expression patterns in both static and compressive conditions for chondrocytes (Figure 4E), which can be explained by the lack of growth factors (*e.g*., TGF-β) in the system, which are often used to stimulate cartilaginous matrix production.^45-48^ Growth factors play a crucial role in chondrogenesis affecting the type of ECM synthesized.^49^ Mauck et al. showed significant upregulation of ECM production when both TGF-β1 and IGF-1 were added as supplements.^50^ Chondrosarcoma cells instead, increased expression of both COL2A1 and COL6A1 in compressive conditions (69% dynamic and 34% static for COL2A1 & 69% dynamic and 17% static for COL6A1) (Figure 4F). The higher expression of COL6A1 observed in chondrosarcoma cells exposed to cyclic compression, compared to static culture may correlate with an increase in tumor aggressiveness, as previously reported by Chen et al.^51^ It is important to underline that we did not see any differences in pericellular matrix formation depending on position of the cells in the hydrogel with respect to the mechanical actuation unit. It would be of interest to investigate whether the molecular mechanisms which are triggered in these two cell types are distinct and/or if their response could be correlated to their membrane stiffness in future studies.

### 3.7. Compressive Forces Impact the Spatial Organization of ACAN, COL1A1 and COL2A1 in Micromass Models

**Figure 5.**
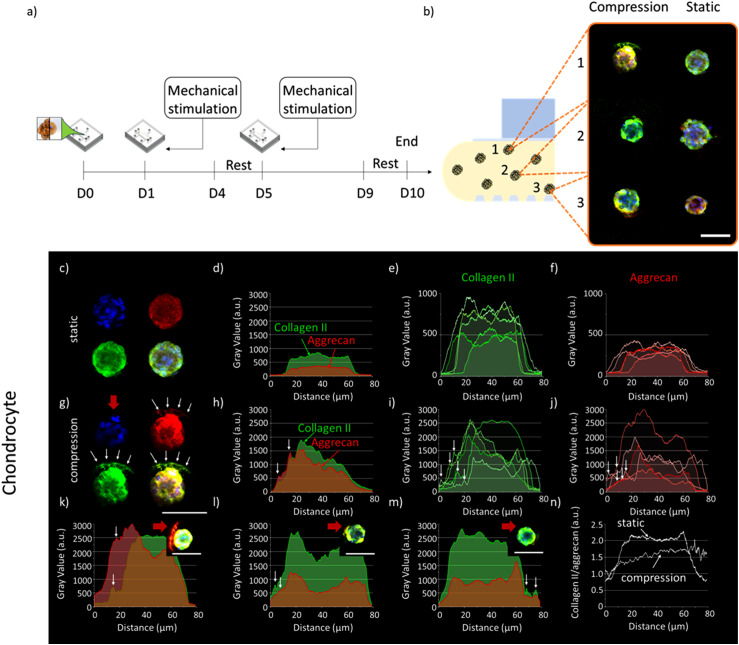
ECM production by chondrocyte and chondrosarcoma micromasses. (A) Schematic summary of the experimental design. (B) ACAN and COL2A1 protein expression in three chondrocyte micromasses in static or compressive culture in the three zones (top, middle and bottom) relative to the actuation unit. Chondrocyte micromasses in static (C) or compressive (G) culture conditions expressing ACAN (red) and COL2A1 (green) and their corresponding histogram showing the spatial distribution of these ECM proteins across these micromasses (D, H). Histogram of COL2A1 (E,I) and ACAN (F,J) spatial distribution for five independent micromasses in the top zone for static (E, F) and compressive (I,J) culture. Histogram of chondrocyte micromasses for ACAN (red) and COL2A1 (green) in the top (k), middle (l) and bottom (m) zones. Arrows in the histogram indicate peaks related to protein protrusion. n) Ratio of COL2A1-to-ACAN spatial distribution for static and compressive culture conditions. Scale bars: 100 µm

In OA, chondrocytes become more proliferative to generate small clusters of cells. Little is known on the mechanisms driving this phenotypic change and associated protein arrangements. We postulate that this behavior could be part of an adaptation mechanism to cope with the hyper-physiological mechanical forces experienced by cells due to cartilaginous matrix deterioration, since micromasses are more resistant to mechanical deformation than single primary chondrocytes, as we demonstrate in this paper (Figure 2). We therefore studied how the differences we observed in ECM protein production in single chondrocytes and chondrosarcoma cells would translate at the micromass level, and if the structural arrangement and production of proteins differs between static and compressive culture conditions (Figure 5A). For these experiments we studied micromasses (n=5) positioned in three zones with respect of the actuation unit (Figure 5B).

**Figure 6.**
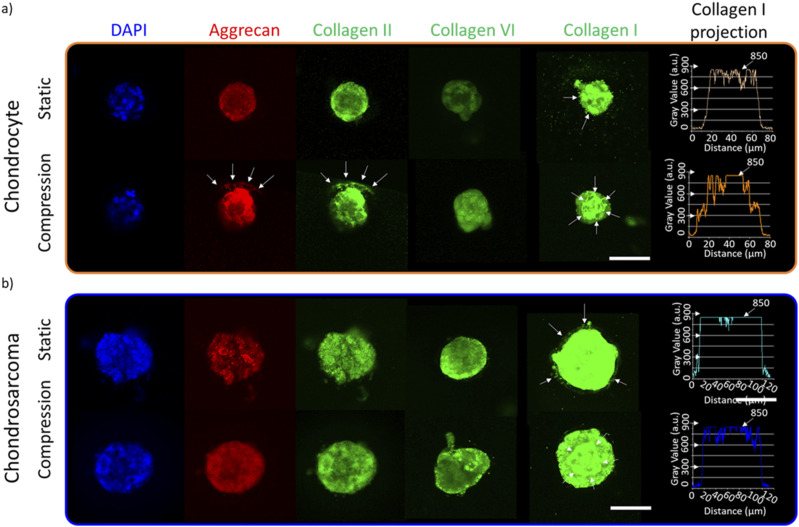
Collagen I core and ACAN production by chondrocyte and chondrosarcoma micromasses. (A) Chondrocyte micromasses expressing ACAN (red) and COL2A1, COL6A1 and COL1A1 (green) in static (top) and compressive culture (bottom). On the left, graph representing the collagen I present within the micromass. (B) Chondrosarcoma micromasses expressing ACAN (red) and COL2A1, COL6A1 and COL1A1 (green) in static (top) and compressive culture (bottom). On the left, graph representing the collagen I present within the micromass. White arrows point to proteins produced in the surrounding of the micromasses which we consider as protein protrusions. The direction of the load is from top to bottom. Scale bars: 100 µm

Exposure to compressive forces was associated with a semi-quantitative trend toward increased ACAN signal in OA chondrocyte micromasses along the loading axis. This descriptive spatial pattern was less apparent in static culture, where ACAN appeared more evenly distributed across the micromass (Figure 5C and F). The total ACAN signal also appeared higher in mechanically stimulated samples compared to static culture, with higher gray values and a lower COL2A1/ACAN ratio (Figure 5F, J and N). This result is in agreement observation is consistent with previous reports on the effect of mechanical cues on single chondrocytes^52^ and in line with the previous section of this paper. A similar trend was observed for COL2A1 deposition, with a formation of a crown of proteins in the direction of load upon application of compressive forces (Figures 5 and 6 & S11). Remarkably, while this protein “crown” was directed towards the actuating membrane in the top zone in the hydrogel, it was absent in the middle zone and directed towards the pillars in the bottom zone (Figure 5 k,l,m).

These semi-quantitative observations suggest, but do not prove, that micromasses may alter ECM deposition patterns in relation to local deformation and position within the device**.** Although an increase in COL2A1 production was already observed at day 4, protein organization was only observed at a later time point (day 10) suggesting that the biomechanical cellular response requires extended period of culture (Figure S5). Because hypertrophic or osteogenic markers were not assessed in this study, possible links to COL10A1, RUNX2, OPN, or catabolic molecules remain speculative and should be investigated in future studies.^3^ ACAN expression was higher in static in comparison to dynamic conditions in chondrosarcoma micromasses (Figure 6B & Figure S7) while COL2A1 was higher upon compression. Interestingly, while ACAN production increased upon prolonged culture, COL2A1 was progressively reduced reaching values lower than at the start of the culture (Figure S7). Our results are partially in line with Palubeckaite et al. who observed an increase in glycosaminoglycan and COL2A1 production in static conditions.^26^ However, the hydrogel, the medium and the duration of the experiment were different compared to our study, and all these parameters are known to influence the behavior of the cells. However, the hydrogel, medium, cell model, and experimental duration differed from those in our study, and all these parameters are known to influence cellular behavior.

We finally examined COL1A1 production in both micromass models. Exposure to compressive forces resulted in an unexpected trend, with accumulation of this ECM protein in the core of both micromasses (Figure 6A-B & Figure S6, 7, 12), irrespective of the micromass location in the hydrogel with respect of the actuation membrane. This COL1A1 core was observed at day 4 for both static and dynamic conditions and for both cell types. However, while in static condition it progressively disappeared over time, upon compression the micromasses maintained this protein organization (Figure S6 & S7). The structural organization of the pericellular matrix in healthy chondrons is known to function as a protective shield for mechanical forces and it acts as a mechano- transducer for chondrocytes.^2^ However, to the best of our knowledge no previous study has investigated protein arrangement in chondrocyte micromasses. At last, no difference was detected in terms of COL6 production for either cell type (Figure 6A-B & Figure S13).

It is unclear why chondrosarcoma cells respond in a different manner than chondrocytes when exposed to mechanical stimulation. It is well known that both cell types present differences in genetic makeup,^53^ stiffness and in integrin responses.^54^ Since integrins are important transducers of mechanical stimuli, these differences may account in part for the differences in the observed cellular responses. Altogether, exposure to compressive forces induces cell type specific responses which are also dependent on the cell’s microenvironment.^55-57^

A limitation of the present study is that it was not designed to directly resolve the signaling pathways responsible for the distinct responses observed between single cells and micromasses or between primary OA chondrocytes (single donor) and CH2879 chondrosarcoma cells. The current data therefore remain primarily phenotypic, based on deformation behavior, gene-expression changes, and semi-quantitative/descriptive ECM organization under cyclic compression. While the differences observed are consistent with organization-dependent and cell-source-dependent mechanosensitivity, we did not directly assess canonical mechanotransduction pathways in this work. In particular, pathways involving YAP/TAZ, integrin-associated signaling such as FAK or ILK, and mechanosensitive ion channels such as PIEZO1/2 may contribute to the responses described here and should be investigated in future studies. Accordingly, our interpretation is limited to the observation that the four models display distinct compression-associated phenotypes within this agarose-based organ-on-chip system, rather than to specific mechanistic conclusions.

## 4. Limitations of the Study

While revealing clear differences in gene and protein expression between chondrocytes and chondrosarcoma cells, and their culture as single cells or micromasses in the OoC device, arguably, the present study still presents some limitations. The use of a 4 mm slab limits the use of microscopes presenting short focal distance hampering the visualization of the construct. However, the cell laden hydrogel constructs can be easily retrieved from the organ-on-chip for high-resolution imaging using confocal microscopy. The comparison of the different Young moduli was based on different techniques which have approaches that may not be comparable to our system. Although remarkable differences in ECM production were observed in our cellular models exposed to cyclic compression compared to static culture, we first acknowledge that the use of LMT agarose does not fully recapitulate the native cellular structural and physical microenvironment^58^ in cartilage or chondrosarcoma. This hydrogel matrix also prevents cell proliferation and may induce possible phenotypic changes by itself. Yet, agarose has been widely used in the past as a matrix for chondrocyte culture.^59-61^ It supports single cell tracking, is free from possible non-specific antibody binding for the analysis of ECM proteins, as agarose only contains sugars. Furthermore, it does not suffer from batch-to-batch variation as is the case for collagen type I and Matrigel^TM^, for instance.

An additional limitation is that the current study was performed in a single agarose formulation and therefore does not distinguish whether the observed compression-associated phenotypes arise primarily from cellular organization itself or from the way this specific hydrogel transmits load to embedded cells and micromasses. In low-adhesive agarose systems, differences in matrix stiffness and poroelastic behavior are expected to influence local deformation, fluid movement, and mechanical cue propagation. Accordingly, the present findings should be interpreted as responses observed under one defined matrix condition rather than as matrix-independent features of cartilage or chondrosarcoma biology. Future studies comparing multiple agarose concentrations or alternative matrix systems will be important to determine how robust the organization-dependent trends are across different mechanical microenvironments.

In our study, we only considered one type of mechanical stimulation (compression at 300 mbar at a 1 Hz frequency for one hour a day) which corresponds to healthy physiological stimulation for cartilage tissue while yielding *in vivo*-like healthy chondrocyte deformation. Modifying the type, frequency and/or amplitude of the stimulation could provide new insights into the behavior of both cells. Stronger mechanical stimulation may alter, either way, the aggressiveness of chondrosarcoma. A combination of compressive and shear stress forces would better recapitulate the stimulation experienced during joint movement, as we previously reported.^19^ This could lead to a better representation of how chondrocyte micromasses respond to a “rolling-like” stimulus such as exposed cartilage in the moving osteoarthritic knee. Nonetheless, by using the multi-directional stimulation model we would reduce the throughput as this platform requires three inlets for the actuation rather than one. Moreover, it could be argued that the difference in cell deformation could result in variations at the transcriptional level. However, the range of stimulation considered in this study overall corresponds to healthy conditions based on cell deformation (Figure 3). It would be of interest to characterize some of the hypertrophic (COL10) and osteogenic markers (RUNX2, OPN) in chondrocyte micromasses upon mechanical stimulation, both at the gene and protein levels.^3,62^ Lastly, our study investigated a single chondrocyte donor and a single chondrosarcoma cell line, which is an important limitation. Hence, to determine whether the effects observed are generally observed a larger number of patients and cell lines should be considered. The results should therefore be interpreted as proof-of-concept observations within a controlled organ-on-chip setting. Both OA cartilage and chondrosarcoma are highly heterogeneous with respect to disease stage, matrix composition, differentiation status, and mechanobiological behaviour. Additional studies including multiple donors and independent chondrosarcoma models will be necessary to determine the robustness and broader relevance of the compression-dependent responses observed here.

## 5. Conclusion

In this proof-of-concept study, we examined the response of primary chondrocytes from one end-stage OA donor and one CH2879 chondrosarcoma cell line to cyclic compressive forces in an agarose-based organ-on-chip platform. We considered two culture formats, single-cell suspensions and small micromasses, both embedded in agarose. Within this specific model, OA chondrocyte micromasses deformed less than single OA chondrocytes, possibly because of compaction and enhanced cell-cell interactions in this 3D configuration. The data further suggest that cyclic compression is associated with model-dependent differences in selected gene-expression and ECM protein readouts among the four culture conditions. ECM spatial patterns in micromasses, including a COL1A1-enriched core and peripheral aggrecan/COL2A1 signal trends along the loading axis, should be interpreted as semi-quantitative descriptive observations rather than evidence of an established mechanism. COL6A1 production did not show a clear response to mechanical stimulation in the micromass models under these conditions. Overall, the main contribution of this study is the establishment of a controlled organ-on-chip framework in which single cells and micromasses from cartilage-derived benign and malignant models can be compared under the same cyclic compression regime while linking deformation, gene-expression changes, and spatial ECM readouts. Within this specific matrix and loading setting, the data support the hypothesis that cell source and culture organization influence compression-associated phenotypes. Broader biological conclusions, however, will require validation in additional donors, cell lines, matrix stiffnesses, and loading regimes.

## Supplemental Material

Supplemental Material - Effects of Compression on Extracellular Matrix Synthesis by Chondrocytes and Chondrosarcoma Cells Supplemental Material for Effects of Compression on Extracellular Matrix Synthesis by Chondrocytes and Chondrosarcoma Cells by Carlo Alberto Paggi, Isa Porsul, Séverine Le Gac, Marcel Karperien in CARTILAGE

## Data Availability

The datasets used and/or analyzed during the current study are available from the corresponding author on reasonable request.*
