# Supplementary material for: Effects of Compression on Extracellular Matrix Synthesis by Chondrocytes and Chondrosarcoma Cells
Source: Cartilage. 2026 Jul 17:19476035261460455. Online ahead of print. doi: 10.1177/19476035261460455 (PMC13379520; doi:10.1177/19476035261460455)
Supplement: Supplemental Material - Effects of Compression on Extracellular Matrix Synthesis by Chondrocytes and Chondrosarcoma Cells [file sj-pdf-1-car-10.1177_19476035261460455.pdf]

Supplemental information

Effects of Compression on Extracellular Matrix Synthesis by Chondrocytes and Chondrosarcoma Cells

Carlo Alberto Paggi, Isa Porsul, Séverine Le Gac, and Marcel Karperien

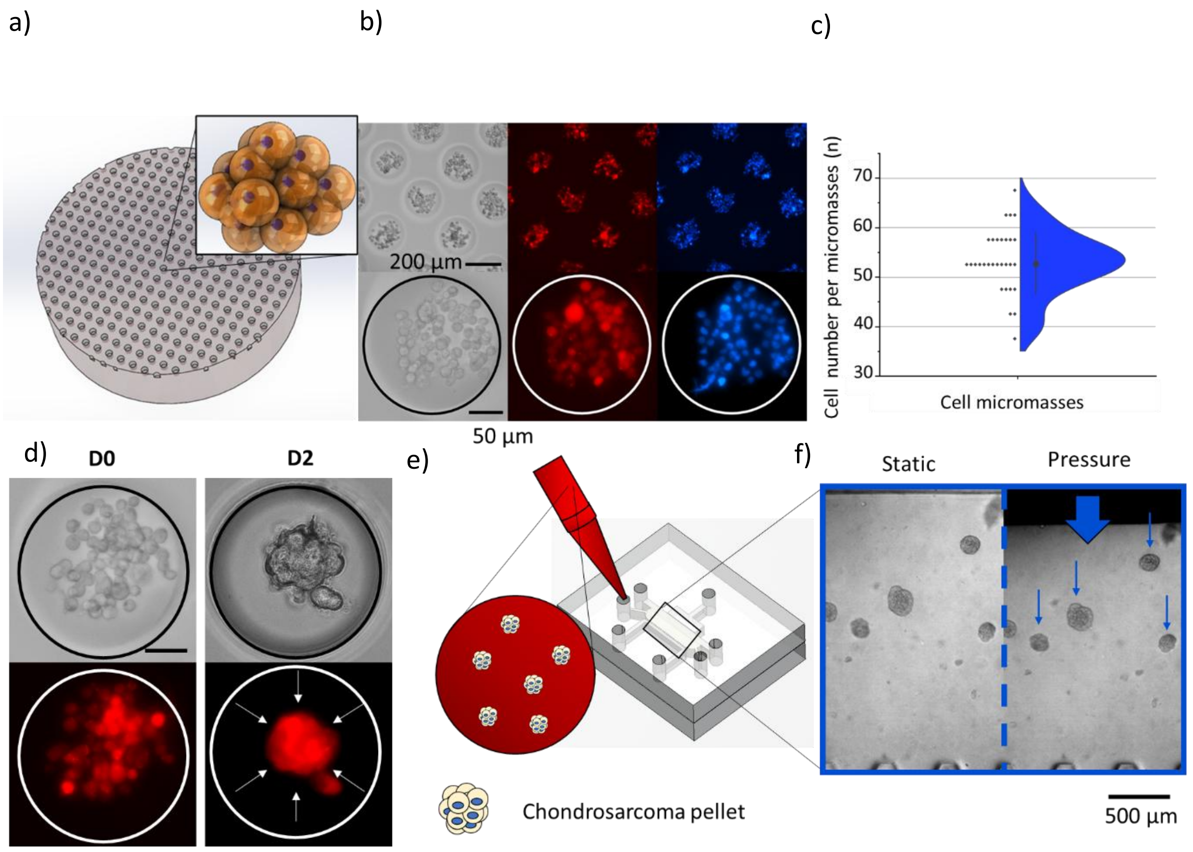

Figure S1: **Overview of the preparation of the micromasses and OoC device with cells**. a) 3D view of the agarose microwell array. b) Top view of the array after seeding of chondrosarcoma cells (transmitted, RFP-positive cells (red), nuclei (DAPI, blue). c) Graph depicting the number of chondrosarcoma cells counted in 30 microwells at day 0 after seeding and before the formation of the micromasses. d) Top view of one microwell with chondrosarcoma cells at day 0 (after seeding and before compaction) and day 2 (after clustering and compaction). e) Schematic representation showing the injection of the micromasses in agarose in the OoC device. f) Top view of an area in the the cell-hydrogel chamber containing chondrosarcoma cell micromasses in static and compressive conditions (500 mbar applied pressure).

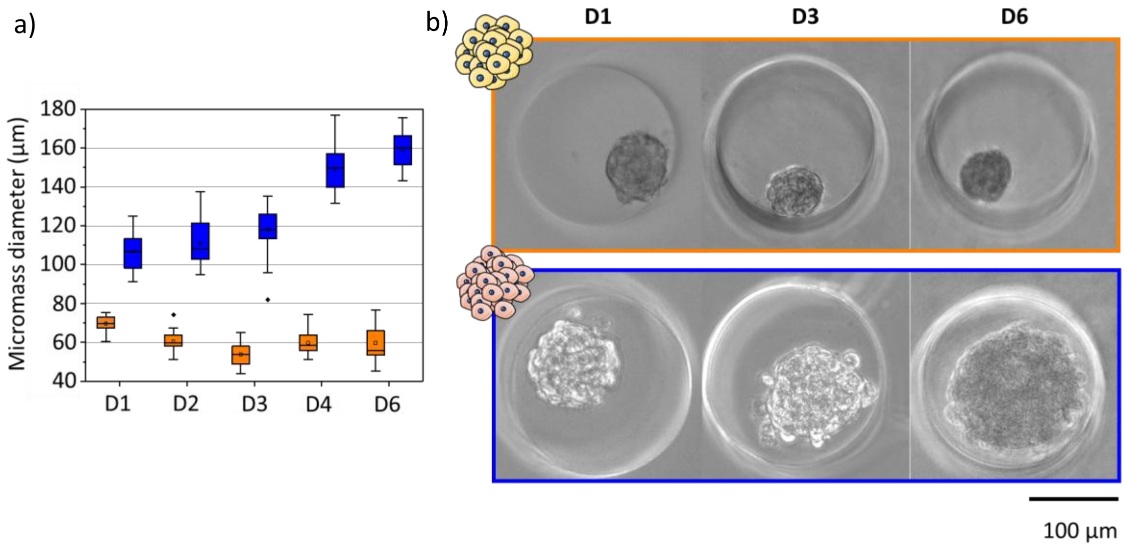

Figure S2: **Size variation of the micromasses**. a) Graph presenting the evolution in the diameter of micromasses based on either chondrocytes (n = 26, orange) and chondrosarcoma *cells* (n =26, blue) as a function of time, when cultured in medium in the agarose microwell array. b) Top view of one agarose microwell presenting chondrocyte (orange frame) or chondrosarcoma cell (blue frame) micromasses at different time points (day 1, 3, 6).

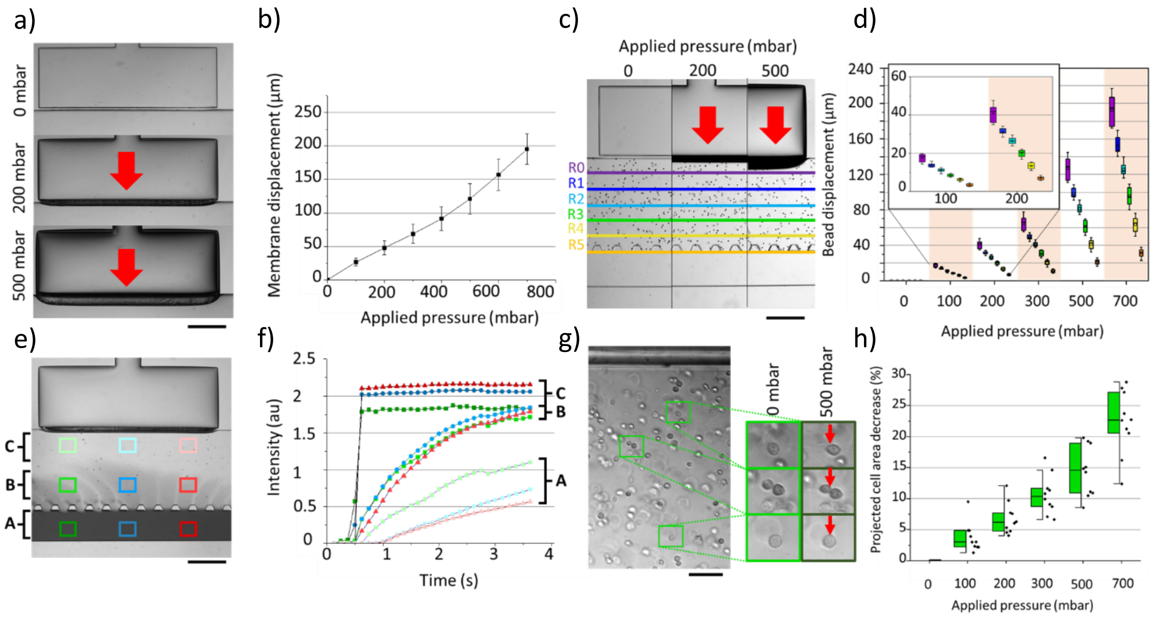

Figure S3: Characterization of the OoC platform. a) Top view of the mechanical actuation unit for different applied pressure levels. Red arrows indicate direction of pressure applied. b) Deformation of the PDMS membrane as a function of the applied pressure, as determined for four independent devices. c) Top view of the actuation unit and hydrogel section filled with agarose supplemented with 15-µm microbeads, showing the subdivision in six zones of 210µm in width; violet (R0), blue (R1), light blue (R2), green (R3), yellow (R4) and orange (R5). d) Microbead displacement as a function of the applied pressure in the six regions, as depicted in (c), using the same color code. e) Top view of the platform showing the nine zones considered to evaluate the delivery of a food dye across the hydrogel section. f) Quantification of the food dye delivery in the nine regions as defined in (e), as a function of time, while applying compressive forces as defined in the text. g) Top view of the platform with chondrocytes embedded as a single-cell suspension in agarose matrix, at rest and under application of compressive forces at different positions in the culture chamber. h) Cell deformation as a function of the applied pressure in the proximity to the membrane. Scale bars for (a, c, e): 500 µm. Scale bar for (g): 100 µm.

Gene name	Forward	Reverse
**GAPDH**	CGCTCTCTGCTCCTGTT	CCATGGTGTCTGAGCGATGT
**SOX9**	TGGGCAAGCTCTGGAGACTTC	ATCCGGGTGGTCCTTCTTGTG
**COL1A1**	GTCACCCACCGACCAAGAAACC	AAGTCCAGGCTGTCCAGGGATG
**ACAN**	AGGCAGCGTGATCCTTACC	GGCCTCTCCAGTCTCATTCTC
**MMP-13**	AAGGAGCATGGCGACTTCT	TGGCCCAGGAGGAAAAGC

Table S1: Human primer sequences for mRNA expression analysis.

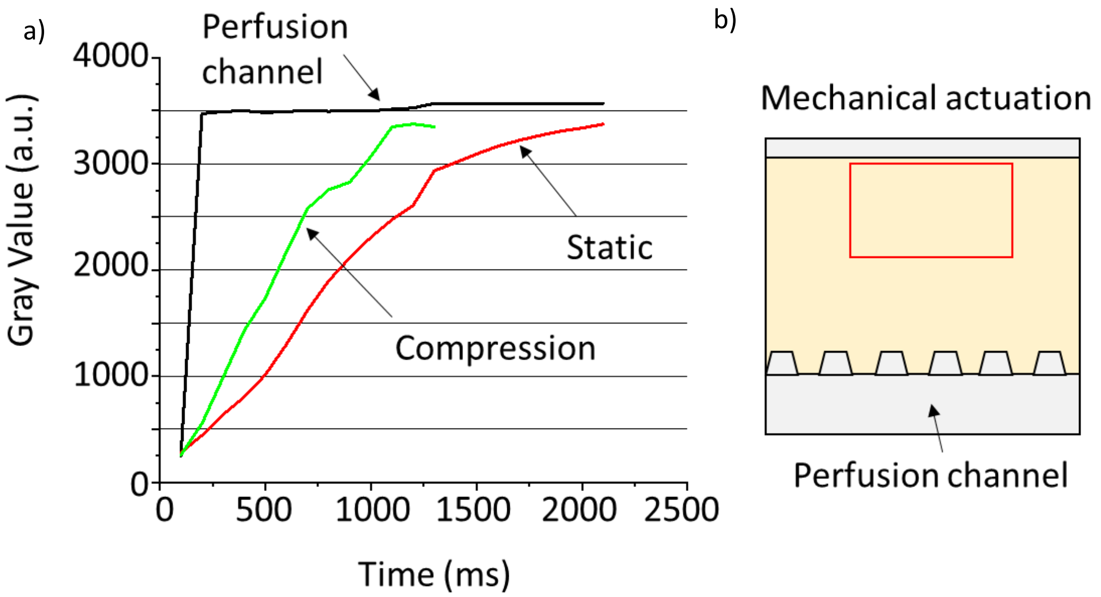

Figure S4: **Diffusion of Rhodamine B in the system in static or dynamic conditions.** a) Graph presenting the intensity of the Rhodamine B in the perfusion channel (black), upon actuation (green) and in static conditions (red). b) schematic of section considered to determine Rhodamine B diffusion.

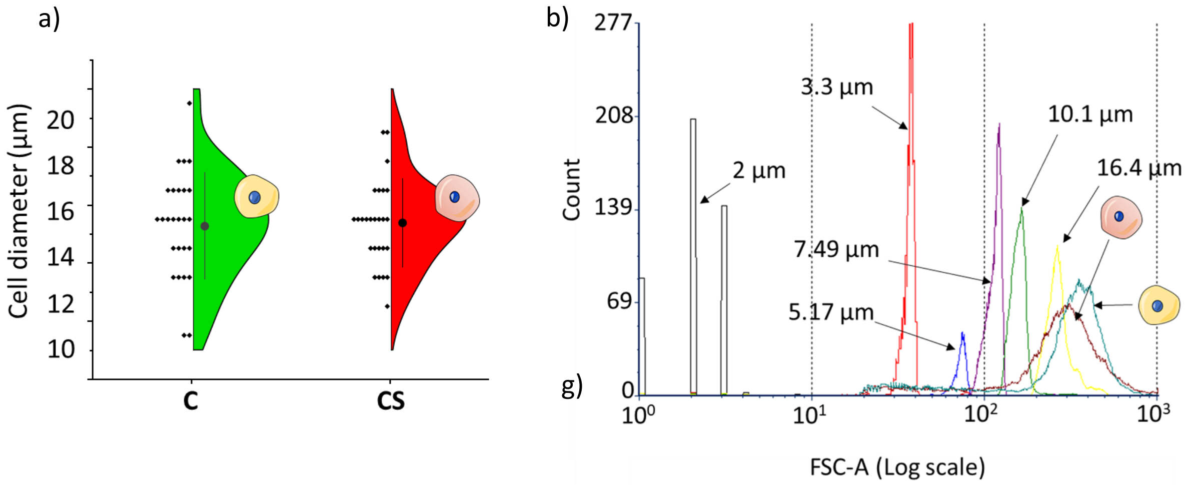

Figure S5: **Cell size characterization.** a) Graph presenting the cell diameter of 26 chondrocytes (green) and 26 chondrosarcoma (red) cells. b) Peaks obtained by flow cytometer while measuring calibration beads (black (2.0 µm), red (3.3 µm), blue (5.17 µm), violet (7.49 µm), green (10.1 µm), yellow (16.4 µm)) and cells (dark red (chondrosarcoma cells) and dark green (chondrocytes)). FSC: forward scatter area.

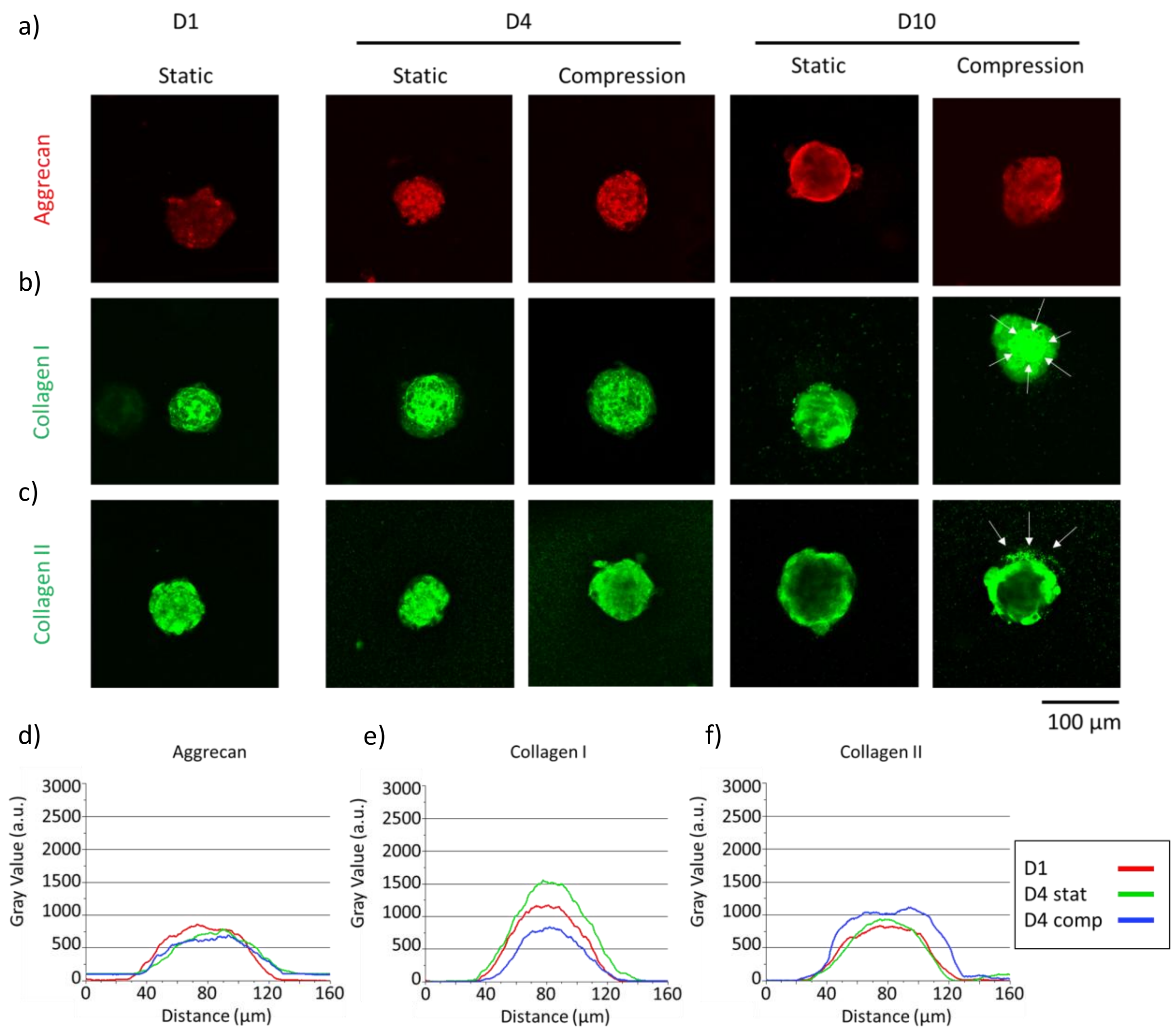

Figure S6: **Chondrocyte micromass protein production.** a) Immunofluorescence at day 1 (left), at day 4 (middle) and day 10 (right) in static and compression conditions of ACAN (a), COL1A1 (b) COL2A1 (c); Histogram of ACAN (d), COL1A1 (e), COL2A1 (f) spatial distribution (n=5 to 8). White arrows indicated protein core or protein protrusion. Scale bar: 100 µm.

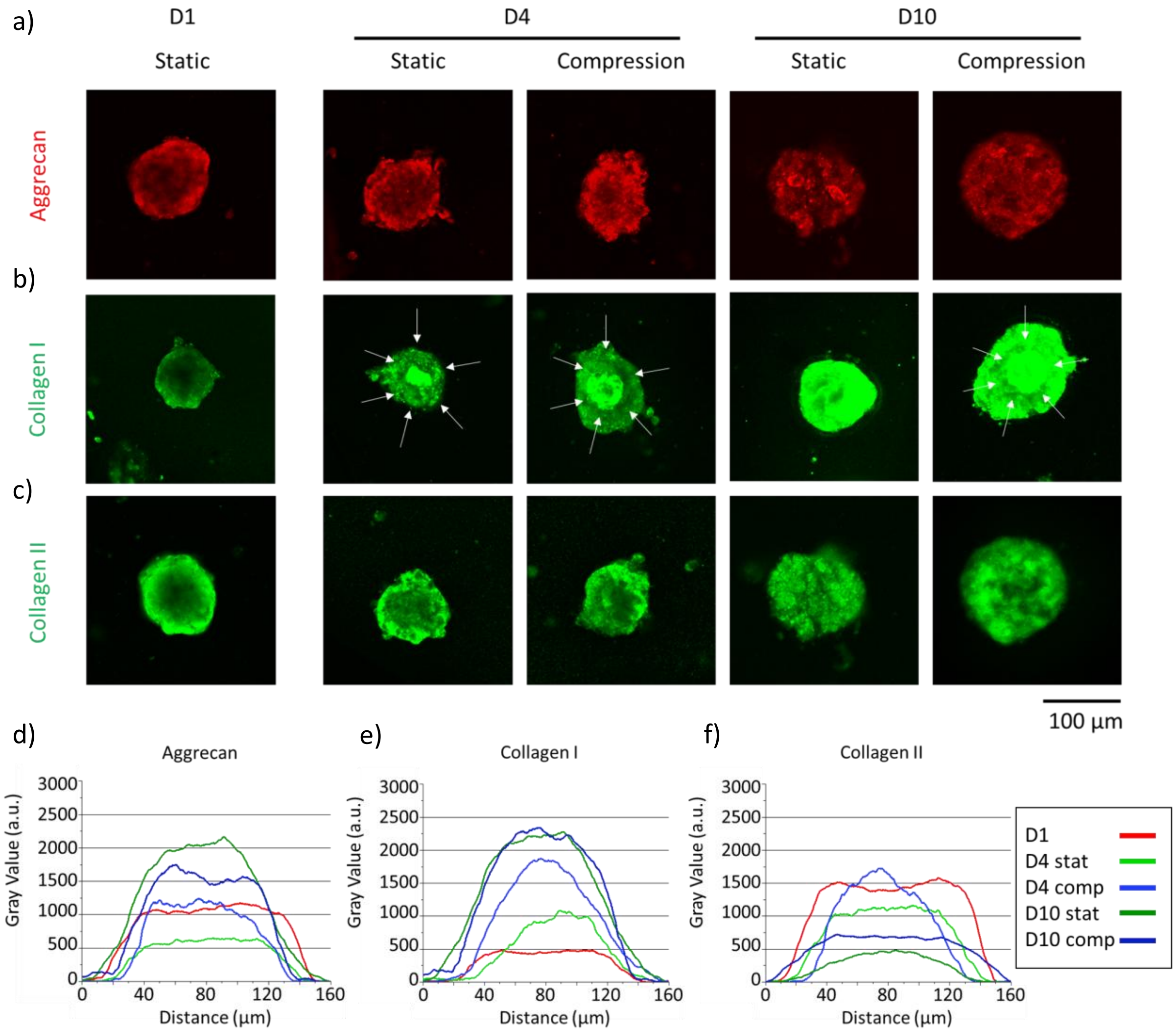

Figure S7: **Chondrosarcoma micromass protein production.** a) Immunofluorescence at day 1 (left), at day 4 (middle) and 10 (left) in static and compression conditions of aggrecan (a) collagen I (b) collagen II (c); Histogram of aggrecan (d), collagen I (e), collagen II (f) spatial distribution (n=5 to 8). White arrows indicate protein core. Scale bar: 100 µm.

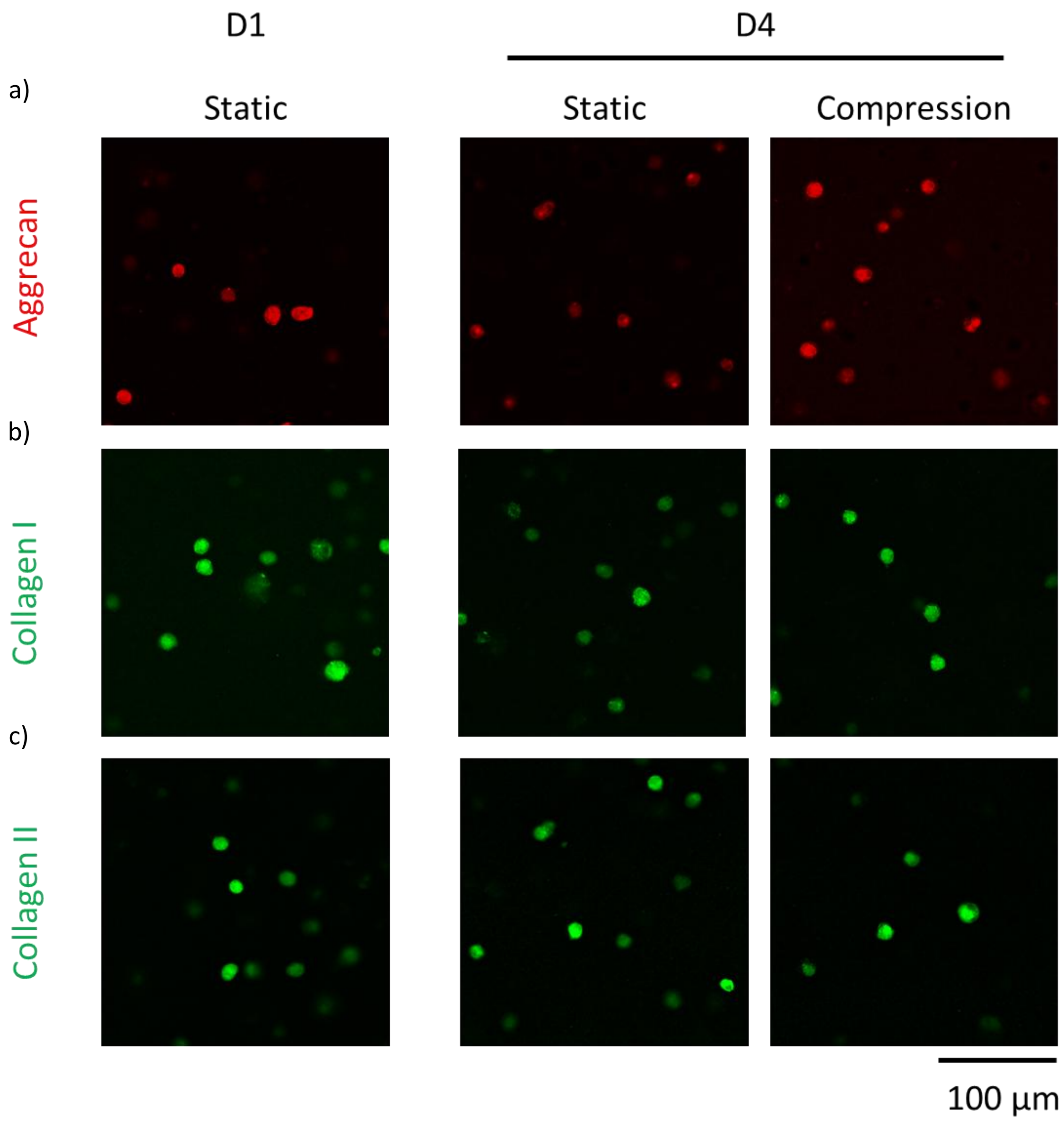

Figure S8: **Chondrocyte single cell protein production.** a) Immunofluorescence of at day 1 (left), at day 4 (middle) in static and compression of aggrecan (a) collagen I (b) collagen II (c). Scale bar: 100 µm.

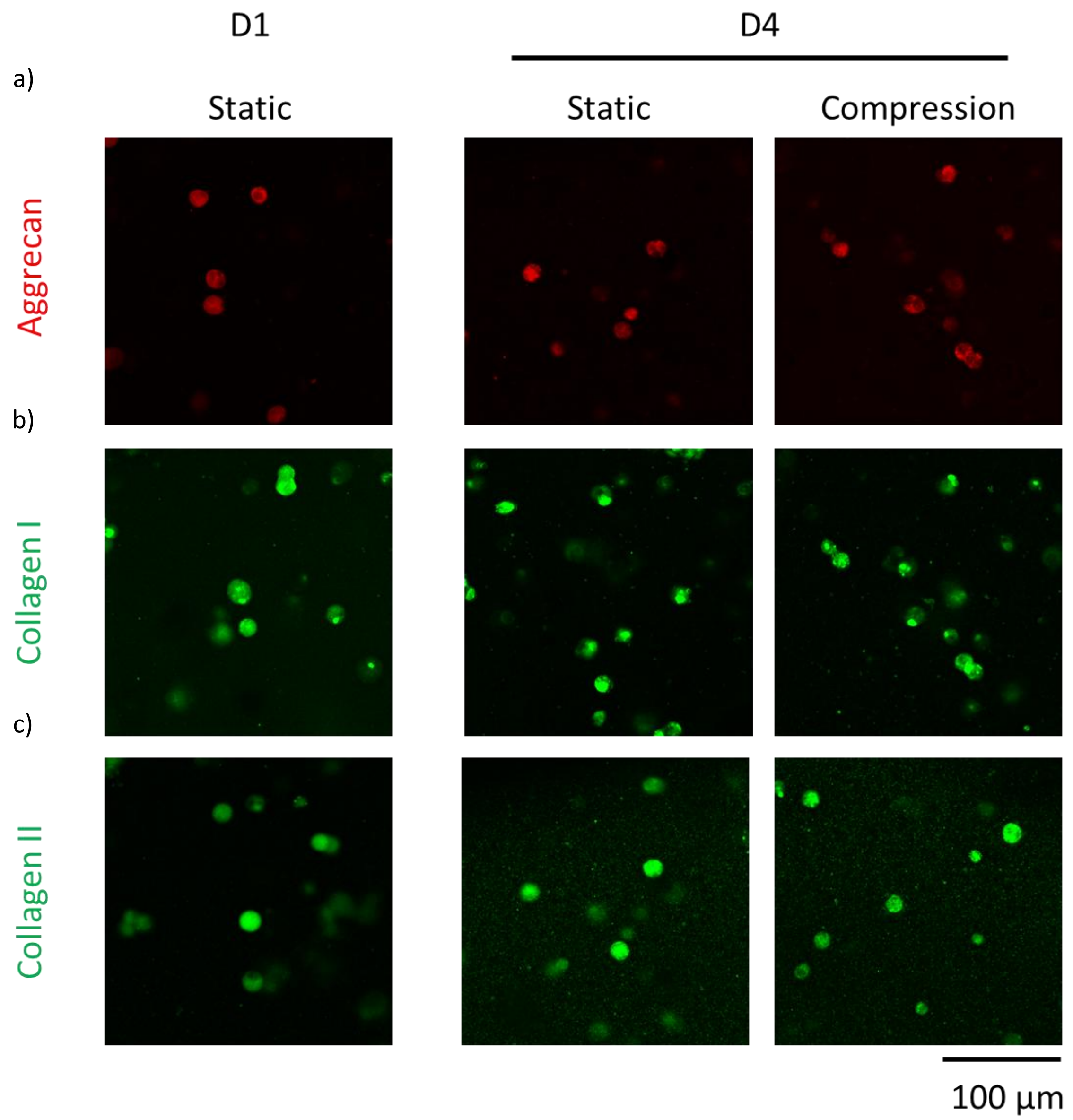

Figure S9: **Chondrosarcoma single cell protein production.** a) Immunofluorescence of at day 1 (left), at day 4 (middle) in static and compression of aggrecan (a) collagen I (b) collagen II (c). Scale bar: 100 µm.

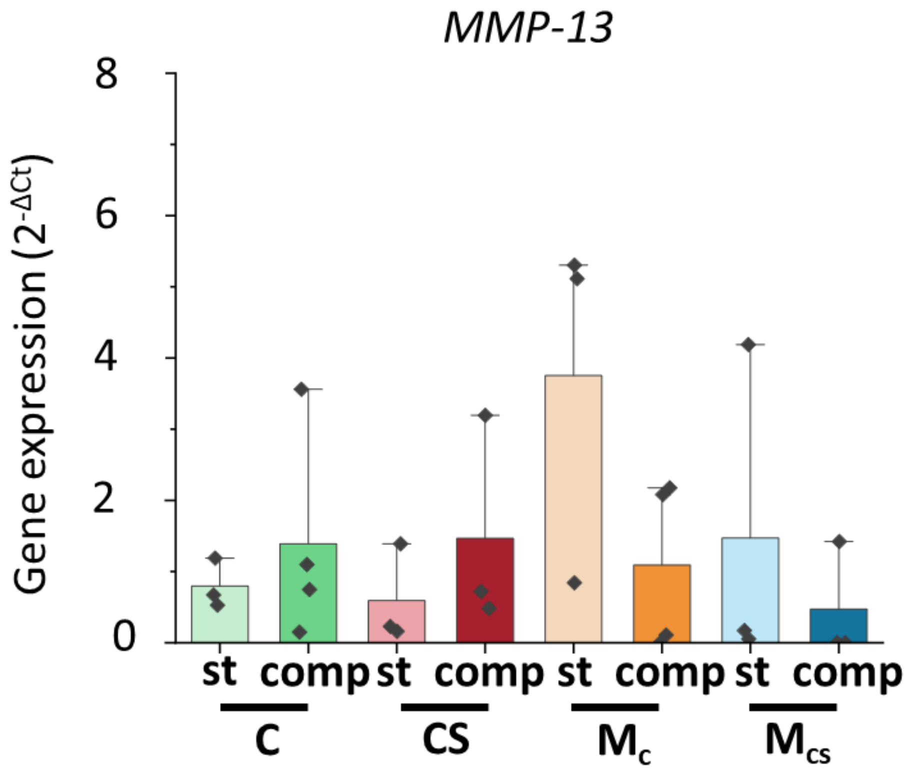

Figure S10: **Gene expression of chondrocyte and chondrosarcoma as single cells and micromasses in static or compression condition**. Gene expression levels of *MMP-13*. Four cellular models are considered: c: single chondrocytes (green), cs: single chondrosarcoma cells (red), Mc: chondrocyte micromasses (orange), Mcs: chondrosarcoma cell micromasses (blue), those models being cultured under static conditions (st) and under compression (comp) as defined in the main article. Data are normalized to the expression of *GAPDH*, as defined in the text.

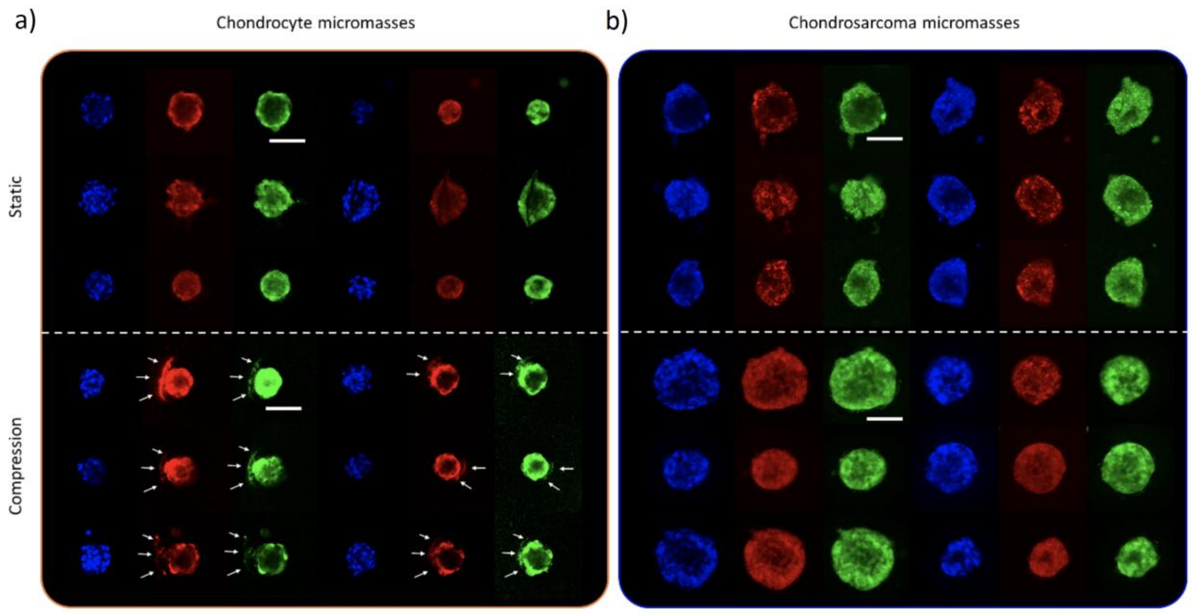

Figure S11: Immunofluorescence of chondrocyte and chondrosarcoma micromasses in static and compression conditions. Extracellular matrix production by chondrocytes (a) and chondrosarcoma cells (b) cultured as micromasses in the organ-on-chip devices in static and compressive conditions (DAPI-blue, aggrecan-red, col II-green). Actuation membrane is at the left side of the figure while pillars and perfusion channel are at the right site. White arrows point towards the formation of an ECM shell by micromasses. Scale bars: 100 µm.

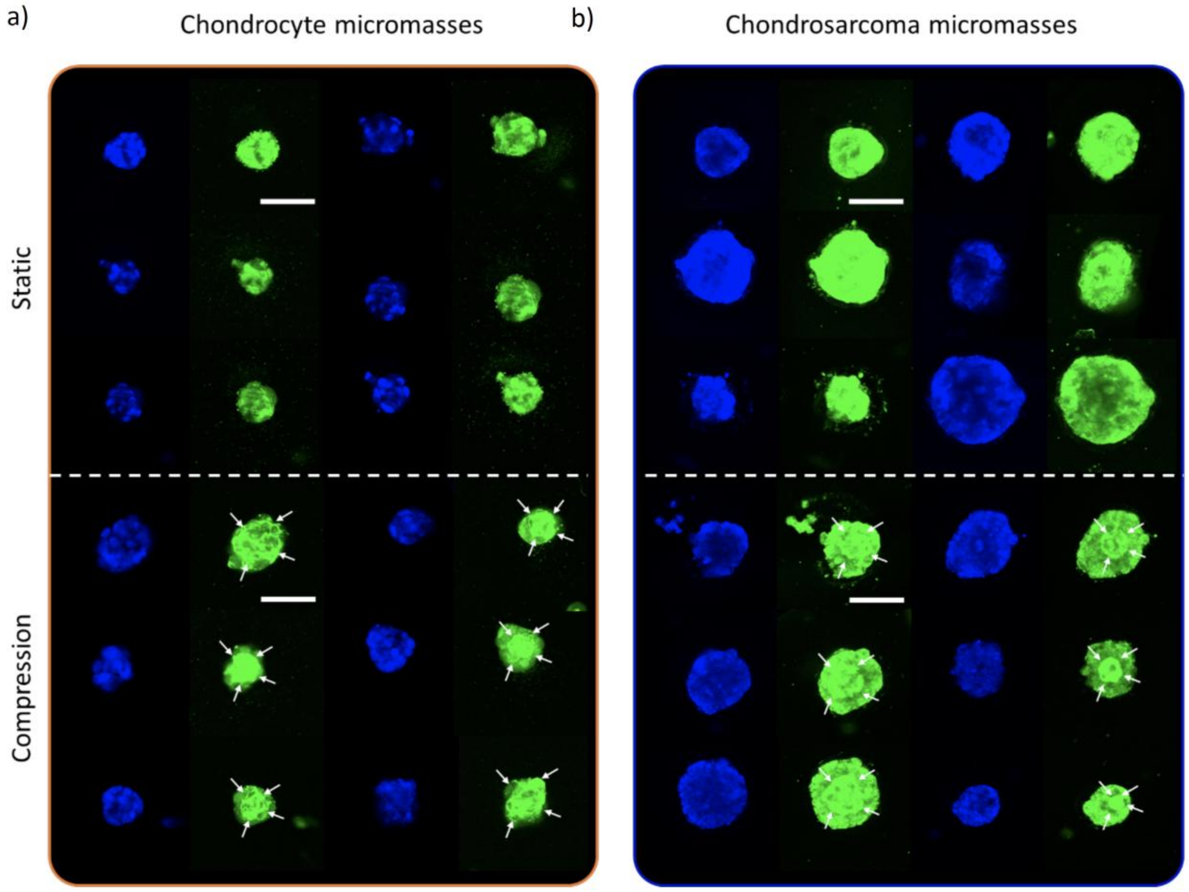

Figure S12: **Immunofluorescence of chondrocyte and chondrosarcoma micromasses in static and compression conditions**. Extracellular matrix production by chondrocytes (a) and chondrosarcoma cells (b) cultured as micromasses in the organ-on-chip devices in static and compressive conditions (DAPI-blue, col I-green). Actuation membrane is at the left side of the figure while pillars and perfusion channel are at the right site. White arrows point towards the formation of an ECM shell by micromasses. Scale bars: 100 µm.

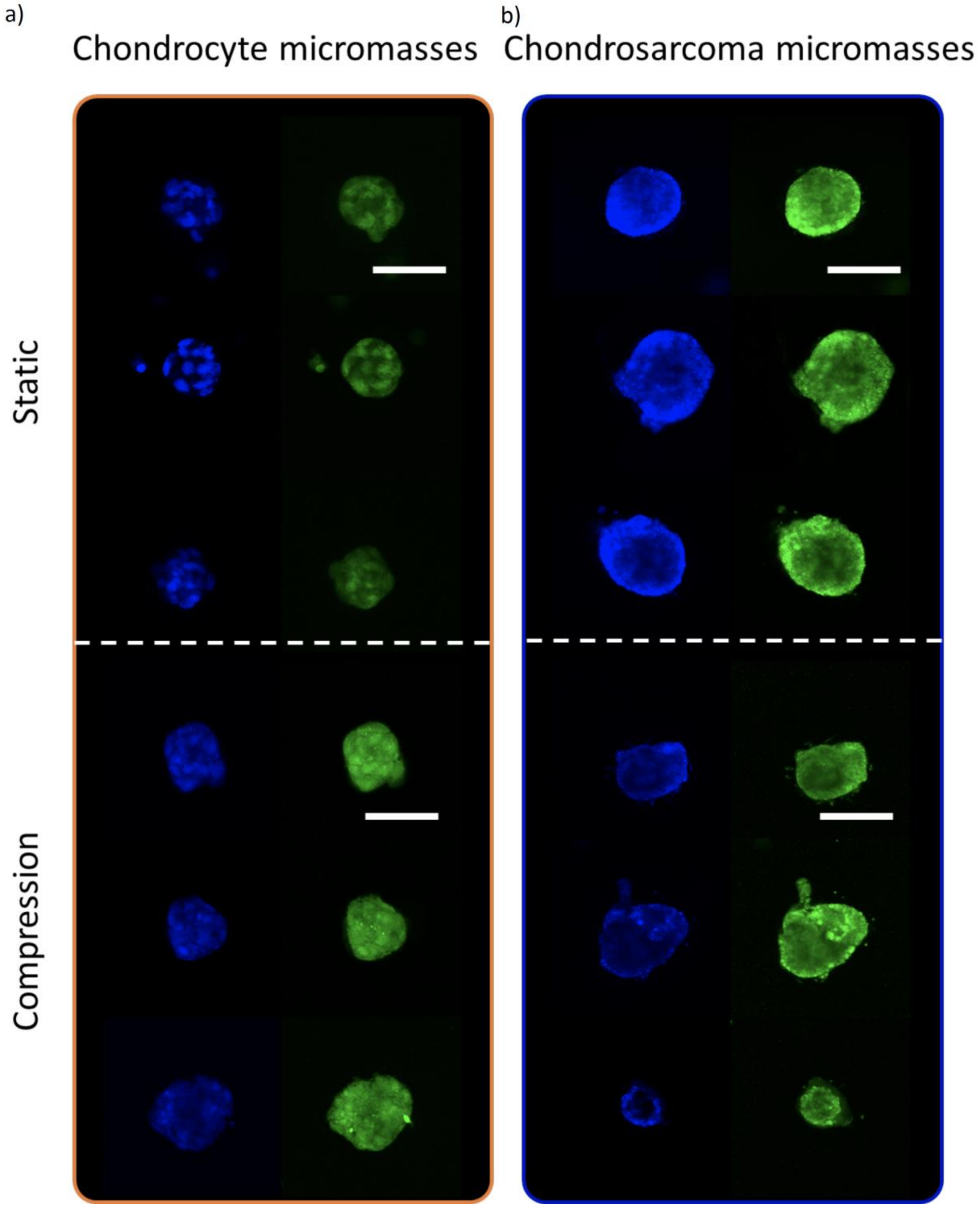

Figure S13: **Immunofluorescence of chondrocyte and chondrosarcoma micromasses in static and compression conditions.** Extracellular matrix production by chondrocytes (a) and chondrosarcoma cells (b) cultured as micromasses in the organ-on-chip devices in static and compressive conditions (DAPI-blue, col VI-green). Actuation membrane is at the left side of the figure while pillars and perfusion channel are at the right site. Scale bars: 100 µm.

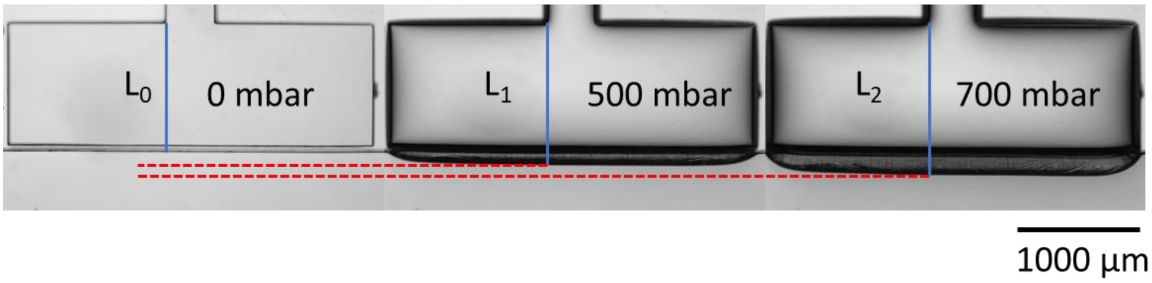

Figure S14: Determination of the menbrane deformation. Top view of the mechanical actuation chamber at rest (left) and under application of compressive forces (500 & 700 mbar, middle & right, respectively), showing how the membrane deformation is determined.

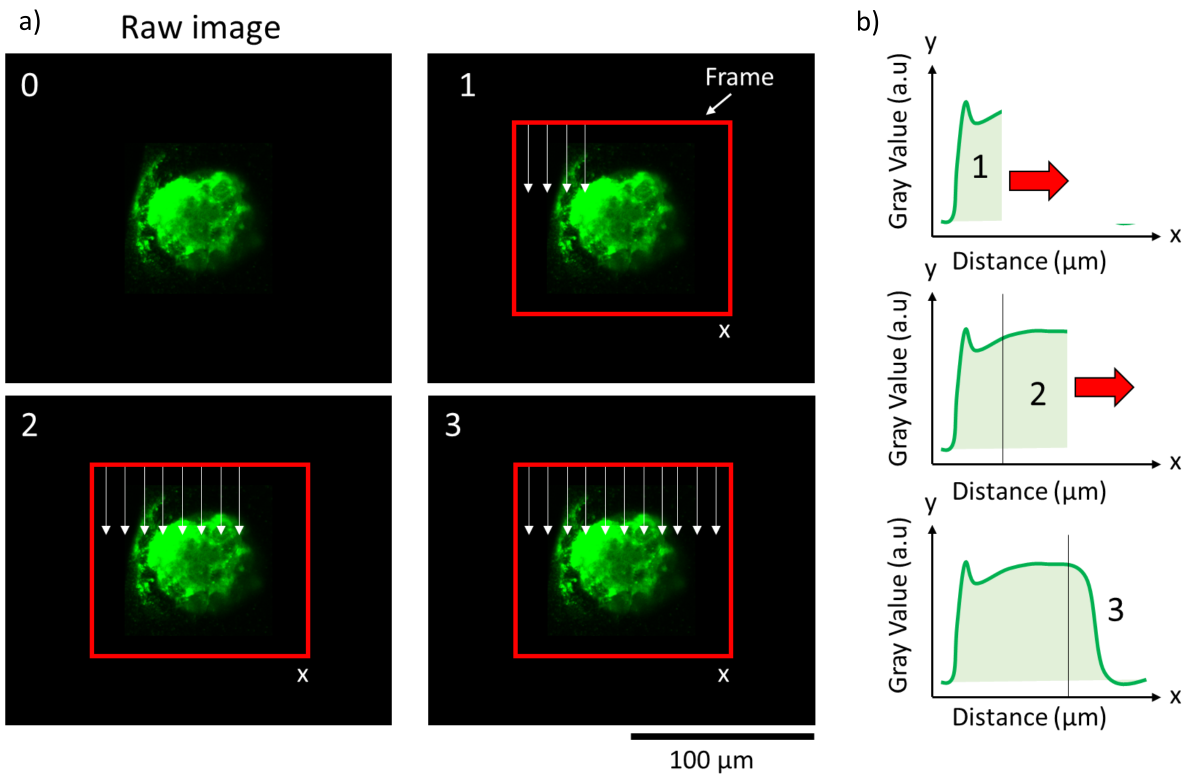

Figure S15: Process of histogram generation. Raw image is uploaded in ImageJ and a rectangle is drawn around the micromass (a). Using a plot function the software generates the average of the pixels present in each column and plots it (b). Scale bar: 100 µm.

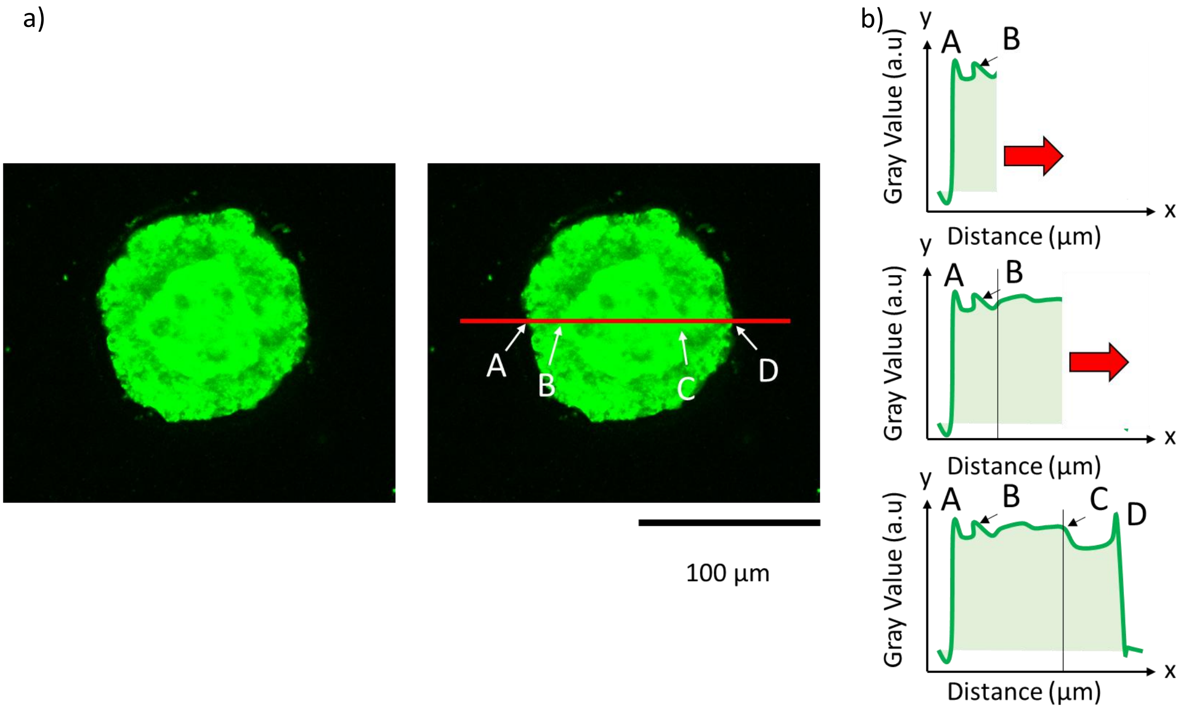

Figure S16: Process of histogram generation. Raw image is uploaded in ImageJ and a line is drawn through the center of the micromass (a). Using a plot function the software generates a plot of the pixel intensity (b). Scale bar: 100 µm.

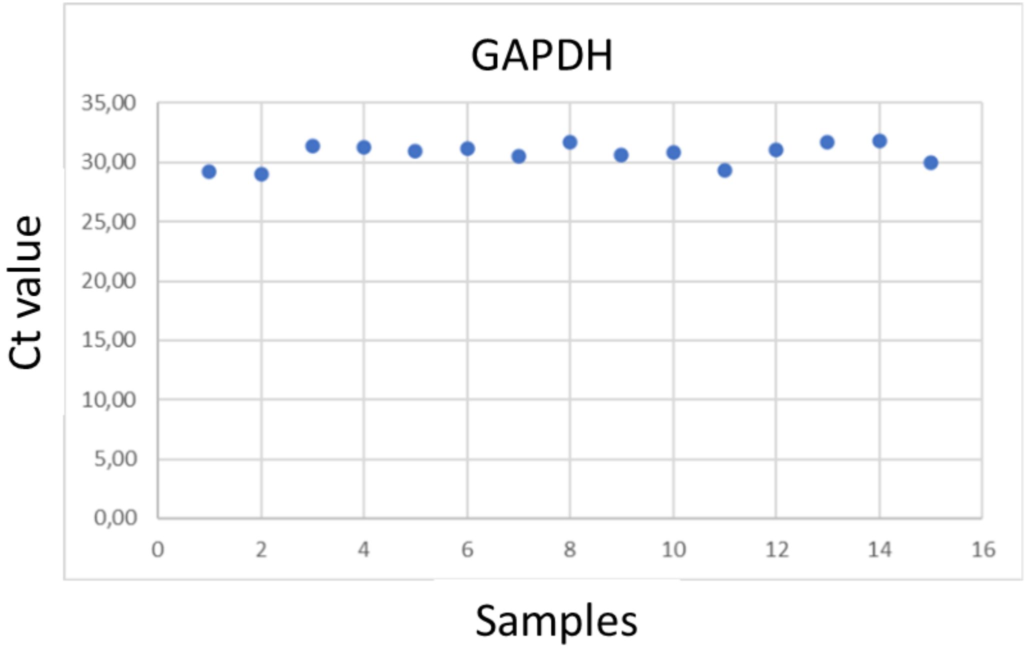

Figure S17: Ct value of GAPDH. Ct values of GAPDH across different samples extracted from cells residing in agarose matrix.
